# The dicot homolog of maize PPR103 carries a C-terminal DYW domain and may have a role in C-to-U editing of some chloroplast RNA transcripts

**DOI:** 10.1007/s11103-024-01424-1

**Published:** 2024-03-15

**Authors:** Tyra N. McCray, Mohammad F. Azim, Tessa M. Burch-Smith

**Affiliations:** 1 School of Genome Science and Technology, University of Tennessee, Knoxville, TN 37996 USA; 2 Department of Biochemistry and Cellular and Molecular Biology, University of Tennessee, Knoxville, TN 37996 USA; 3 Donald Danforth Plant Science Center, St. Louis, MO 63132 USA

**Keywords:** Chloroplast gene expression, C-to-U editing, DYW domain, *Nicotiana benthamiana*, Pentatricopeptide repeat, PPR103, RNA editing

## Abstract

In plants, cytidine-to-uridine (C-to-U) editing is a crucial step in processing mitochondria- and chloroplast-encoded transcripts. This editing requires nuclear-encoded proteins including members of the pentatricopeptide (PPR) family, especially PLS-type proteins carrying the DYW domain. *IPI1/emb175/PPR103* is a nuclear gene encoding a PLS-type PPR protein essential for survival in *Arabidopsis thaliana* and maize. Arabidopsis IPI1 was identified as likely interacting with ISE2, a chloroplast-localized RNA helicase associated with C-to-U RNA editing in Arabidopsis and maize. Notably, while the Arabidopsis and *Nicotiana* IPI1 orthologs possess complete DYW motifs at their C-termini, the maize homolog, ZmPPR103, lacks this triplet of residues which are essential for editing. In this study we examined the function of IPI1 in chloroplast RNA processing in *N. benthamiana* to gain insight into the importance of the DYW domain to the function of the EMB175/PPR103/ IPI1 proteins. Structural predictions suggest that evolutionary loss of residues identified as critical for catalyzing C-to-U editing in other members of this class of proteins, were likely to lead to reduced or absent editing activity in the *Nicotiana* and Arabidopsis IPI1 orthologs. Virus-induced gene silencing of *NbIPI1* led to defects in chloroplast ribosomal RNA processing and changes to stability of *rpl16* transcripts, revealing conserved function with its maize ortholog. *NbIPI1*-silenced plants also had defective C-to-U RNA editing in several chloroplast transcripts, a contrast from the finding that maize PPR103 had no role in editing. The results indicate that in addition to its role in transcript stability, NbIPI1 may contribute to C-to-U editing in *N. benthamiana* chloroplasts*.*

**Supplementary Information:**

The online version contains supplementary material available at 10.1007/s11103-024-01424-1.

## Key message

The *Nicotiana benthamiana* DYW PPR protein NbIPI1 possess an intact C-terminal DYW domain and stabilizes the *rpl16-rpl14* transcript like its maize ortholog PPR103, and may also contribute to C-to-U RNA editing of some chloroplast transcripts.

**Supplementary Information:**

The online version contains supplementary material available at 10.1007/s11103-024-01424-1.

## Introduction

In land plant organelles, post-transcriptional processing of RNA transcripts is a crucial regulatory point for gene expression. One step of post-transcriptional RNA processing is the site-specific deamination of cytidine to uridine, called C-to-U editing. In most land plants this C-to-U RNA editing occurs within a subset of chloroplast and mitochondrial transcripts. Editing within these transcripts often produces changes in splice sites, amino acid substitutions and the addition of start and stop codons. These modifications yield transcripts that can subsequently be translated to produce proteins essential for photosynthesis and for mitochondrial function (Small et al. 2020).

C-to-U editing involves numerous nucleus-encoded proteins including several families of RNA binding proteins. The largest family of plant proteins with roles in post-transcriptional processing and C-to-U editing of organelle transcripts is the pentatricopeptide repeat (PPR) protein family. PPR proteins are site-specific RNA-binding proteins that have critical and diverse functions (Saha et al. 2007; Schmitz-Linneweber and Small 2008; Barkan and Small 2014; Small et al. 2020). Loss of a single PPR protein can result in embryonic arrest or severe developmental defects, demonstrating their fundamental importance to plant survival and development (Barkan and Small 2014). Plant PPR proteins are typically targeted to either the mitochondria or chloroplast, where they act by binding to one or several single-stranded RNA molecules via 2–30 N-terminal tandem helical repeat motifs (Shikanai 2006). Within plant mitochondria and chloroplasts, most characterized PPR proteins mediate specific events in post-transcriptional processing and maturation of RNA by influencing RNA splicing, RNA cleavage, RNA stability, translation and the site-specific sequence alteration of RNA transcripts through a process called RNA editing [reviewed in (Barkan and Small 2014)]. Other cellular processes that are affected by PPR proteins include nuclear gene expression (Ding et al. 2006; Koussevitzky et al. 2007; Liu et al. 2010) and plastid biosynthesis (Beick et al. 2008).

PPR proteins are divided into the P subfamily and the plant-specific PLS subfamily, and both classes of PPR proteins have diverse roles in RNA metabolism. In *Arabidopsis thaliana*, the P subfamily comprises approximately half of the 450 known PPR proteins. While the P family proteins contain only PPR repeats, the PLS subfamily is further subdivided according to the domains present in the extended C-terminal regions of subfamily members: (i) E proteins contain an E domain as the C-terminal region (ii) E + proteins carry an E domain and an E + C-terminal region, and (iii) DYW proteins possess an E, E + and an additional domain named the DYW domain due to the presence of aspartic acid, tyrosine, and tryptophan triplet of amino acids (DYW), or a variation thereof, at their extreme C-termini (Shikanai 2006). The DYW domain appears to be plant-specific and has sequence similarity to cytidine deaminases (Salone et al. 2007; Iyer et al. 2011), enzymes involved in the recognition of target cytidines in the C-to-U editing reaction (Okuda et al. 2014). The PPR DYW domain has been correlated with the occurrence of RNA editing, while the E domains are thought to recruit editing enzymes (Schmitz-Linneweber and Small 2008). PPR proteins within the PLS subfamily with known functional roles in RNA editing all belong to the E or DYW PPR subfamilies (Shikanai 2006).

The RNA editing factor interacting proteins (RIP)/Multiple organellar RNA editing factor (MORF) proteins are a small family of proteins required for C-to-U editing in both chloroplasts and mitochondria (Takenaka et al. 2012; Bentolila et al. 2013). RIP/MORF2 and 9 are required for editing of chloroplast transcripts while the other proteins are involved in mitochondrial transcript editing (Takenaka et al. 2012; Tian et al. 2019). RIP1/MORF8 is dual-targeted to chloroplasts and mitochondria (Bentolila et al. 2012). RIP/MORF proteins carry a novel conserved domain of about 100 amino acids, the MORF box, that is required for multimerization and interaction with PPR proteins (Takenaka et al. 2012; Bayer-Csaszar et al. 2017; Haag et al. 2017; Yan et al. 2017; Yang et al. 2018). MORF proteins interact with PPR proteins (Bentolila et al. 2012; Takenaka et al. 2012; Glass et al. 2015; Yan et al. 2017) to induce structural changes that increase RNA-binding and editing efficiency (Yan et al. 2017). While PPR proteins are usually involved in editing of a few specific sites, loss of some RIP/MORF proteins can cause defects in editing of all sites in chloroplasts (RIP/MORF2 and 9) (Takenaka et al. 2012) or hundreds of sites in mitochondria (Bentolila et al. 2012). Biochemical evidence has been interpreted as indicating that the organellar RNA editing machinery likely includes an RNA helicase component (Takenaka and Brennicke 2003; Hegeman et al. 2005). Editing activity in chloroplast extracts could be stimulated by ATP, CTP or dCTP (Hegeman et al. 2005) and similarly, mitochondrial extracts from pea that were used for in vitro editing assays could use any NTP or dNTP (Takenaka and Brennicke 2003). Consistent with the proposed involvement of an RNA helicase in organelle RNA editing, the chloroplast RNA helicase ISE2 was shown to be required for editing of several different Arabidopsis chloroplast transcripts (Bobik et al. 2017). The involvement of ISE2 in chloroplast C-to-U editing is supported by the identification of the maize ISE2 orthologue in RIP9 complexes purified from maize extracts (Sandoval et al. 2019), indicating that ISE2 is involved in editing in multiple plants. In studies to determine ISE2’s involvement in chloroplast RNA processing, we identified protein partners of ISE2 including a DYW protein encoded by *At5g03800/EMB175* as a potential partner of ISE2; we subsequently named this DYW protein ISE2 PROTEIN INTERACTOR1 (IPI1) (Bobik et al. 2019; Ganusova et al. 2020). Arabidopsis *embryo defective 175* (*emb175*) mutant embryos arrest at the globular-heart transition (Cushing et al. 2005). The maize ortholog of IPI1/EMB175, PPR103, functions in rRNA processing and stabilization, and loss of PPR103 resulted in seedling lethality (Hammani et al. 2016).

While ZmIPI1/PPR103 lacks the C-terminal triplet of amino acids characteristic of DYW proteins and was reported to not have a role in chloroplast C-to-U RNA editing, NbIPI1 and its dicot orthologs have a DYW motif. In this study, sequence and structural analysis revealed that NbIPI1 and several of its orthologs lack critical residues known to be essential for C-to-U RNA editing. Our modeling predictions suggested that these proteins likely lack a complete coordination site for a critical zinc ion that is required for catalysis of editing. We investigated the function of NbIPI1 in plants where *NbIPI1* was silenced by virus-induced gene silencing (VIGS) in *Nicotiana benthamiana*, bypassing the lethality of the *emb175/ipi1* Arabidopsis mutants (Cushing et al. 2005). Analysis of chloroplast RNA transcripts revealed that PPR103/IPI1 functions in rRNA processing and stabilizing *rpl16* transcripts were partially conserved in *N. benthamiana*. In contrast to maize PPR103, *NbIPI1-*silenced plants had reduced although not completely disrupted C-to-U editing of 23 sites in 18 chloroplast transcripts. These findings suggest that NbIPI1 may have functions related to C-to-U editing although it may not directly function in deamination catalysis.

## Materials and methods

### Plant materials and growth conditions

*Nicotiana benthamiana* seedlings were grown on a light cart at 25 °C under fluorescent white light in a 16:8-h light/dark cycle. Ten-day-old seedlings were transplanted to individual pots and typically silenced using VIGS at around 2–3 weeks of age.

### Transient expression and confocal microscopy

Leaves of five to six-week-old plants were agroinfiltrated with constructs for expression of AtIPI1-YFP, cTP-AtIPI1-YFP or cTP-NbIPI-YFP. Forty-eight hours later infiltrated leaf sections were vacuum infiltrated with water and mounted on slides for imaging. Confocal fluorescence microscopy was performed using a Leica SP2 or SP8X confocal laser scanning microscope (Leica Microsystems, Heidelberg GmbH). A 40 × or 63 × HCX PL APO objective was used for image acquisition. The samples were excited with an excitation line of 458/514 nm for YFP.

### VIGS constructs and protocol

Tobacco rattle virus (TRV)-based VIGS constructs used for the non-silencing control and silencing *ISE2* and *IPI1* were described previously (Burch-Smith and Zambryski 2010; Ganusova et al. 2020). Constructs were transformed into *Agrobacterium tumefaciens* GV3101 (pMP90RK) strain. VIGS of *IPI1*, *ISE2*, *GUS* intron (negative control) and *PDS* (positive control) were performed according to (Burch-Smith and Zambryski 2010; Ganusova et al. 2020). Approximately 3-week-old *N. benthamiana* plants were infiltrated with a mixture of Agrobacterium strains containing TRV RNA1 (pYL192) and TRV RNA2 (pYL56) containing the silencing constructs, and then grown under standard growth conditions until downstream assays were performed 14 days later.

### Transmission electron microscopy (TEM)

Samples were prepared for TEM as described previously (Burch-Smith and Zambryski 2010; Burch-Smith et al. 2011). Briefly, samples from young emerging leaves from control or *IPI1-*silenced plants were fixed by high-pressure freezing (HPF) and quick freeze substituted (QFS) in 1% osmium tetroxide plus 0.1% uranyl acetate in acetone. Subsequently, samples were embedded in epoxy resin Embed 12, (Ted Pella, Inc., Redding, CA), sliced into ultrathin 65–70-nm sections, and visualized on a Libra 200 M TEM/STEM (Carl Zeiss Microscopy, White Plains, NY) at 200 kilovolts.

### Structural modeling

We carried out structure prediction and modeling of the C-terminal portions of NbPPR103/IPI1 using the ColabFold implementation of AlphaFold2 (ColabFold v1.5.2, AlphaFold2_mmseqs2; (Mirdita et al. 2022). We selected residues 757–890 of NbIPI1 sequence as the input sequence. AlphaFold2 was run with the “use PDB templates” option set to “true” and instructed to output its top 5 models after Amber relaxation. Most other interface options were kept at their default values. The top 5 output models were of uniformly high predicted confidence (average plddt were extremely similar to each other [RMSd < 0.15 Å]). We therefore used the top-ranked model by plddt score for subsequent inspection and further modeling.

We inspected the structures and generated molecular graphics using PyMol v.2.50 (The PyMol Molecular Graphics System, Version 2.0, Schrödinger, LLC). We specifically carried out structural comparisons with a number of template homologous domain structures as references to identify and manually adjusted putative Zn^2+^ binding sites within the modeled NbIPI1 C-terminal domains. The homologous experimental structures included fragments and domains of *Arabidopsis thaliana* OTP86 (PDB 7O4E, 7O4F; Takenaka et al. 2021), *A. thaliana* DYW1 [PDB 7W86; (Toma-Fukai et al. 2023)], and AlphaFold pre-predicted structures of *A. thaliana* and *A. lyrata* EMB175/IPI1 (alphafold.ebi.uk entries Q9FFN1 and D7LWU0). Where appropriate, we added and manually adjusted Zn^2+^ ions and water molecules to the NbIPI1 model within PyMol and adjusted nearby sidechains using backbone-dependent rotamers.

### Prediction of NbIPI1 binding sites

Potential binding sites for NbPPR103/IPI1 were predicted with the FIMO program in the MEME suite (Grant et al. 2011). The nucleotide-binding probabilities for NbPPR103/IPI1 were generated based on the amino acids found at the 6 and 1’ position (first amino acid of the subsequent C-terminal PPR motif) of each PPR motif to assign a nucleotide preference according to the weighting scheme in (Takenaka et al. 2013). These nucleotide preferences scores were used to predict NbPPR103/IPI1 RNA binding sites within the *Nicotiana benthamiana* chloroplast genome using the FIMO program. The ten top-predicted binding sites were ranked by *P-*values calculated by FIMO (Grant et al. 2011).

### Northern blotting

Total RNA was run on a denaturing formaldehyde gel, transferred to positively charged Roche nylon membranes (MilliporeSigma, Burlington, MA), and hybridized with DIG-labeled 5S, 23S rRNA and *rpl16* probes (Table S1) according to manufacturer’s instructions (PCR DIG Probe Synthesis Kit, Roche). The amounts of RNA we used for each blot are indicated in the figure legends. Bands corresponding to ribosomal RNA species were detected using the Roche DIG High Prime DNA Labeling and Detection Starter Kit II (MilliporeSigma, Burlington, MA). The same RNA that was used to measure C-to-U editing efficiencies was used for the Northern Blot analysis.

### Chloroplast isolation and RNAseq library preparation

We extracted chloroplasts according to “Extraction of Chloroplast Proteins from Transiently Transformed *Nicotiana benthamiana* Leaves” bio protocol (Klinkenberg 2014; Klinkenberg et al. 2014). Briefly, fresh leaf tissue was ground, filtered and centrifuged through a Percoll gradient and visualized on an inverted microscope. Chloroplasts were then shock-frozen and total RNA was isolated from purified chloroplasts using Trizol (Thermo Fisher Scientific, Waltham, MA) or RNeasy Plant Mini kit (Qiagen, Germantown, MD) as per manufacturers’ instructions. For each plant, we ground approximately 100 mg of tissue from each leaf to isolate chloroplast RNA. Leaves from individual plants were pooled. Removal of chloroplast DNA was done by treating the samples with Ambion rDNase1 (Thermo Fisher Scientific, Waltham, MA). Because rRNA typically constitutes over 75% of total RNA and its depletion can results in very low yields of RNA for cDNA preparation, rRNA depletion was not performed. The RNA integrity of the isolated RNA was examined on a Bioanalyzer machine and quantitated on a NanoDrop 1000 spectrophotometer (Thermo Scientific, Waltham, MA) prior to library preparation. For cDNA synthesis, about one microgram of non rRNA-depleted RNA was used to make double strand cDNA (ds-cDNA) and dsDNA was produced using the Invitrogen SuperScript II Double Stranded cDNA Synthesis kit (Thermo Fisher Scientific, Waltham, MA) with random hexamers primers for first-strand synthesis. The cleaned ds-cDNA was then used to construct a library using the Illumina Next Tera Library prep kit with no adaptations (Illumina, Inc, San Diego, CA). After examination of the library quality using the Bioanalyzer (Agilent, Santa Clara, CA), multiplexed libraries were sequenced using the Illumina MiSeq sequencing platform per standard MiSeq run parameters (Illumina protocol manuals).

### Mapping and data statistical analysis

We examined sequence reads for sequence quality, trimmed using the base space graphical user app (Base Space, Illumina, Inc) and mapped to the *N. benthamiana* genome (NC) using DNA Array Star Next Gen Seq software (version 12) permitting multiple mismatches to detect multiple SNPs. We used the mapping parameters kmer size of 21 and low SNP filter stringency (to avoid missing highly edited transcripts). Paired-end mapped contigs were visualized in Seq Man Pro software or the Integrative Genomics Viewer (IGV) software. The SeqMan NGen-mapped contigs (Supplementary Table S2) were used for subsequent analysis. The uniquely mapped reads were used to detect coverage information for each sample. The coverage summary additionally reveals the depth of sequenced reads that were mapped at each locus in the *Nicotiana* genome. Overall, a similar number of reads were mapped to the *N. benthamiana* genome in all samples.

### RNA editing by sanger sequencing

For editing in the non-silenced control, *ISE2-*, *IPI1-* or *PDS*-silenced leaves, RNA was isolated from leaf number 11 of approximately six-week-old plants using Trizol (Thermo Fisher Scientific, Waltham, MA). The RNA was treated with DNase (30 min with 0.5 μL rDNase, 15 min of a 2nd 0.5 μl rDNase at 37 °C) at least once. RT-PCR was conducted according to manufactures instructions in the M-MLV RT (Promega, Madison, WI,) manual using random primer hexamers. A typical reaction consisted of PCR: 1 μg RNA, 1.2 μL random hexamer, 0.8 μL reverse transcriptase. The same reaction without the reverse transcriptase was performed in parallel with the experimental cDNA synthesis reaction to ensure the absence of genomic DNA contamination. Second-strand PCR synthesis was performed according to standard Taq polymerase protocol using primers in Table S1. PCR was conducted with an annealing temperature of 53 for almost all primer pairs. PCR products were gel purified using the Gel Extraction Wizard kit (Promega, Madison, WI). Purified amplicons were sequenced by the UTK Genomics Core Facility.

### Measuring editing efficiency

RNA was isolated using RNEasy Plant Mini Kit (QIAGEN, Germantown, MD). QPCR was conducted using SYBR Select Master Mix (Applied Biosystems, Thermo Fisher Scientific, Waltham, MA) and primers listed in Table S2, following our previously published protocol (Ganusova et al. 2020) and using the CFX384 Touch Real Time PCR Detection System (BIO-RAD, Hercules, CA).

## Results

### The PPR protein, NbIPI1, is needed for chloroplast development in N. benthamiana

**Fig. 1:**
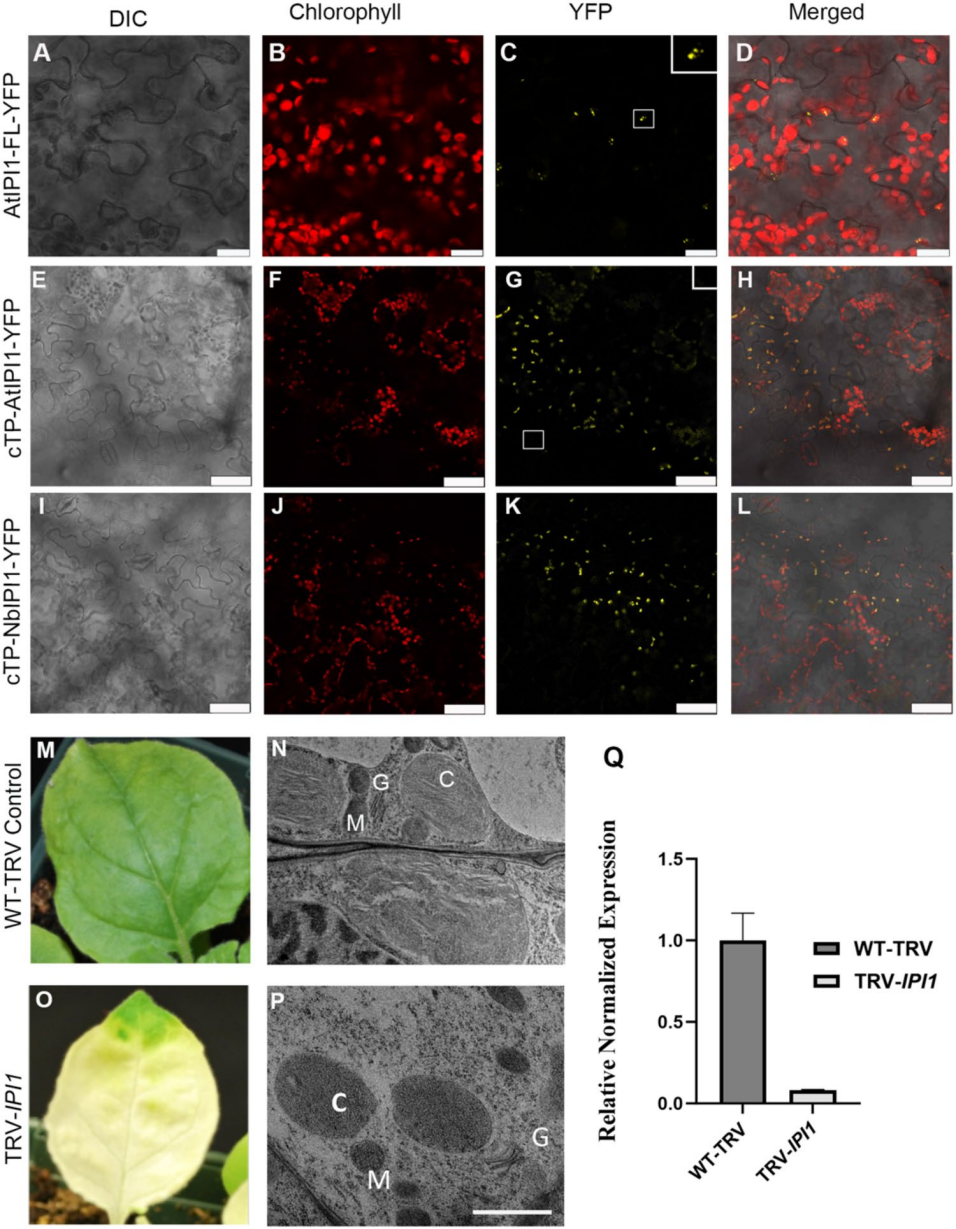
Subcellular localization of IPI1 and effects of *NbIPI1* knockdown on leaves and chloroplasts. **a**–**d** AtIPI1-YFP was transiently expressed in *N. benthamiana* leaves and YFP signal overlapped with chloroplast autofluorescence. Scale bars represent 20 µm. Inset shows enlarged image of boxed region. **e**–**f** The predicted cTP of AtIPI1 was fused to YFP and transiently expressed in *N. benthamiana* leaves. Inset shows enlarged image of boxed region. Scale bars represent 50 μm. **g**–**h** The predicted cTP of NbIPI1 was fused to YFP and transiently expressed in *N. benthamiana* leaves. Scale bars represent 50 μm. **m**–**n** TRV-infected, non-silenced control leaves, and TEM image showing chloroplasts in young sink leaves with forming thylakoids and grana. C, M, and G indicate chloroplast, mitochondria, and Golgi, respectively. **o**–**p** *IPI1*-silenced leaves presented a severe chlorotic phenotype and TEM analysis reveals defective chloroplasts. Scale bar represents 1 μm. **q** Silencing efficiency was measured by quantitative PCR. Statistical significance was determined by Student t test. ***p < 0.001

We previously identified the PPR protein encoded by *At5g03800/emb175/IPI1* as interacting with the chloroplast RNA helicase ISE2 in a yeast two-hybrid screen (Bobik et al. 2019). To determine the subcellular localization of IPI1, we cloned the full-length Arabidopsis *IPI1* coding sequence upstream of the yellow fluorescent protein (YFP). The resulting fusion protein (AtIPI1-YFP) was transiently expressed in *N. benthamiana* leaves and visualized by confocal laser scanning fluorescence microscopy. Fluorescence from the IPI-YFP fusion colocalized with chloroplast autofluorescence, indicating that AtIPI1-YFP localized to chloroplasts (Fig. 1a–d). AtIPI1-YFP also localized to punctae within the chloroplast, suggesting that it may localize to the chloroplast stroma. A similar pattern of fluorescence was observed when the predicted chloroplast targeting peptide (cTP) plus 20 amino acids downstream of the cTP were cloned as a translation fusion to YFP (cTP-AtIPI1-YFP; Fig. 1e–h). To determine NbIPI1’s subcellular localization, a similar construct carrying the predicted cTP of NbIPI1, cTP-NbIPI1-YFP, was transiently expressed in *N. benthamiana* leaves. Fluorescence from cTP-NbIPI1-YFP co-localized with chloroplast autofluorescence, indicating that the fusion was imported into chloroplasts and that NbIPI1 localizes to the chloroplast stroma (Fig. 1–l). Unfortunately, we were unable to clone full-length NbIPI1 to test its subcellular localization despite numerous attempts. Based on our results with the cTP-NbIPI1-YFP construct, we conclude that it is likely that NbIPI1 localizes to chloroplasts given that its chloroplast targeting peptide is functional, although localization to additional compartments cannot be ruled out.

Arabidopsis *emb75* mutants fail to develop past the very early stage of embryonic development, arresting at the globular embryo stage (Cushing et al. 2005). We therefore used VIGS to silence *NbIPI1* in young *N. benthamiana* plants and examine NbIPI1’s function. VIGS of *NbIPI1* in *N. benthamiana* caused severe leaf chlorosis and reduced chlorophyll content [(Fig. 1m, o and q); (Ganusova et al. 2020)]. Transmission electron microscopy on young sink leaves from silenced plants revealed profound effects of *IPI1* knockdown on chloroplast development. While thylakoids and nascent grana were observed in TRV-infected non-silenced controls (Fig. 1n), these structures were largely absent from chloroplasts in *NbIPI1*-silenced leaves (Fig. 1p). These observations are consistent with localization of NbIPI1 to chloroplasts (Fig. 1a–l) and severe chlorosis of *NbIPI1*-silenced leaves (Fig. 1o) and suggest that NbIPI1 has critical roles in chloroplast development.

### IPI1 orthologues have divergent sequences hinting at differing functions

**Fig. 2:**
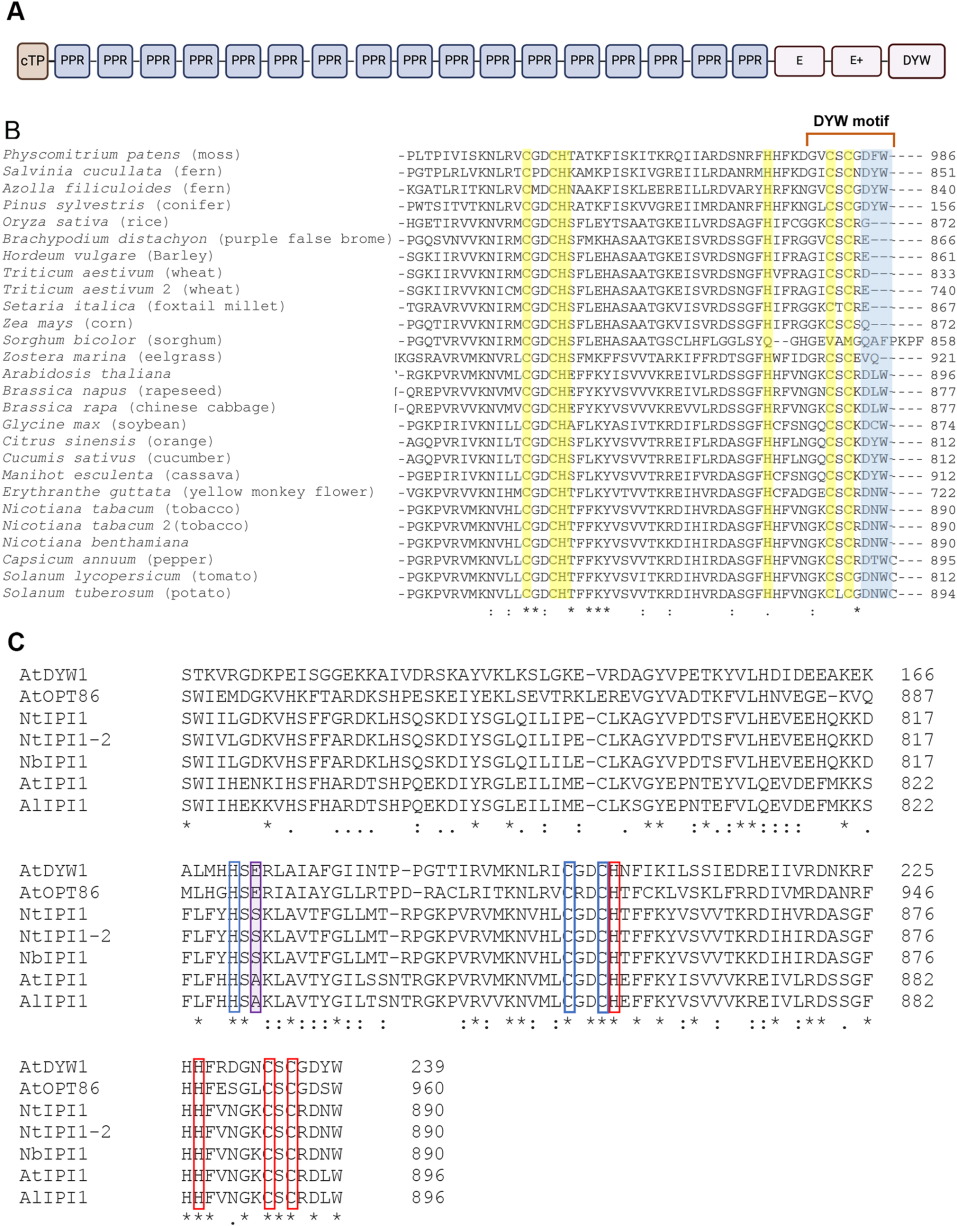
PPR103/EMB175/IPI1 domain structure and residues important for editing. **a** The predicted domains (UniProt) of AtIPI1 including a chloroplast targeting peptide (cTP), 17 PPR domains, an E-domain (E), an E+ domain (E+),  and a DYW domain (DYW). **b** Amino acid sequence alignment of the extreme C-terminal regions of select NbPPR103/IPI1 orthologs. DYW triplet is shown in blue shading. Asterisks, colons, and periods indicate identical amino acid residue, conserved substitution, and semi-conserved amino acids, respectively. Zn^2+^ coordinating residues are shaded in yellow. **c** Alignment of the putative DYW domains of *N. benthamiana*, *A. thaliana* and *A. lyrata* IPI1 sequences with the defined DYW domains of A. thaliana DYW1 and OPT86. Coordinating residues for the catalytic and structural Zn^2+^ binding sites are respectively outlined in blue and red. The catalytically important glutamic acid residue of canonical DYW domains is highlighted in purple

NbIPI1 is predicted to comprise a plastid-localization sequence at its N-terminus, 13 PLS PPR motifs and C-terminal E and DYW domains (Fig. 2a and Supplementary Figure S1). The DYW domain is named after the C-terminal aspartate, tyrosine and tryptophan sequence that is often found in PPR proteins associated with C-to-U RNA editing in organelles (Gutmann et al. 2020).

IPI1 orthologues from early land plants including mosses and ferns also contain a so-called DYW motif consisting of 9 C-terminal residues (Takenaka et al. 2021) (Fig. 2b). The DYW motif is also present in dicots represented in the alignment (Fig. 2B and Supplementary Figure S1). In these sequences, the tyrosine residue is most often substituted by leucine as in Arabidopsis (DLW) or asparagine as in *N. benthamiana* (DNW). Interestingly, the C-terminal DYW triplet is absent from the DYW motifs of the examined monocots, as has been reported for maize [Fig. 2b, (Hammani et al. 2016)]. This finding is supported by phylogenetic analysis, which shows that the monocot proteins form a distinct clade (Supplementary Figure Fig. S2). The absence of the DYW motif from the maize IPI orthologue, PPR103, likely explains previous observations that suggest that maize PPR103 is not involved in C-to-U RNA editing of maize chloroplast transcripts (Hammani et al. 2016).

Recent findings revealed that a canonical DYW domain catalyzes the C-to-U editing through a novel mechanism in which a catalytic zinc atom is regulated by its coordination state (Takenaka et al. 2021). In all orthologues we examined, almost all the residues involved in Zinc binding (highlighted in yellow) are highly conserved (Fig. 2b and c). An important exception is a key glutamic acid residue (E894 in OTP86) within a gating subdomain that is essential for catalysis (Boussardon et al. 2014; Hayes et al. 2015; Oldenkott et al. 2019) (Fig. 2c, highlighted in purple). This glutamate residue, which coordinates the catalytic zinc ion, is substituted with serine in NbIPI1 and with alanine in Arabidopsis EMB175/PPR103 proteins (Fig. 2c).

### Structural prediction of IPI1 structures

**Fig. 3:**
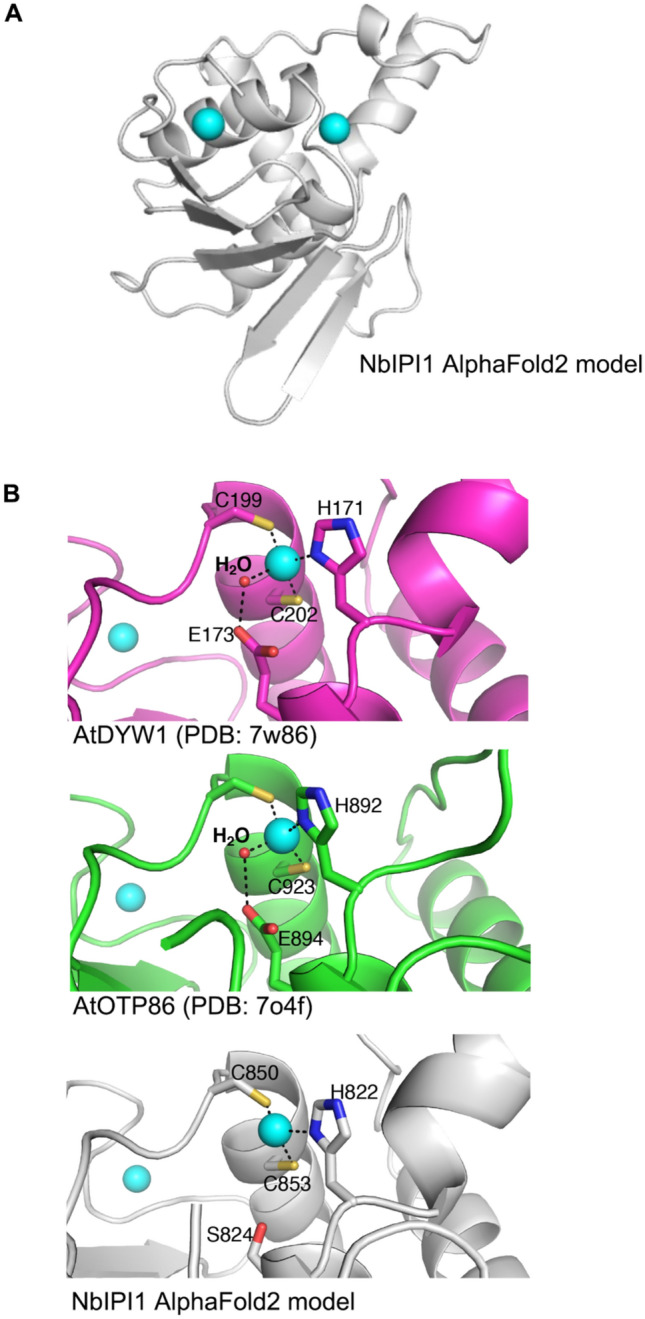
Structural modeling of the NbIPI1 DYW domain. **a** AlphaFold2 model of the C-terminal sequences of NbIPI1, comprising the DYW domain and corresponding to residues 757–890 of the full-length protein. Cyan spheres indicated modeled Zn^2+^ ions. **b** Closeups of the catalytic Zn^2+^ binding site of *A. thaliana* DYW1 and OTP86 (top and middle panels; PDB 7W86, 7O4F) showing the full coordination of the zinc ion (cyan sphere), including a coordinating bound water molecule (small red sphere). The sidechain of the key catalytic glutamic acid residue is shown in each panel. The lowest panel shows the equivalent region of the NbIPI1 model. The NbIPI1 serine that occupies the site of the key glutamic acid cannot effectively substitute for the glutamate as a water-binding residue nor directly coordinate the Zn^2+^ ion

To follow up on the amino acid sequence analysis and get a better understanding of possible NbIPI1 functions, we used the protein structure prediction package AlphaFold2 to generate structural models of the DYW domain of NbIPI1 and compare them with the protein structures of DYW1 (PDB:7W86) or the DYW domain of OTP86 (PDB: 704F). Based on these comparisons, NbIPI1 appears to contain two Zn^+2^ ion binding sites similar to those of canonical DYW domains, one of which participates in catalysis in such DYW domains (Fig. 3a and Supplementary Figures S3 [model of whole DYW domain] and S4 [structural Zn^+2^ binding domain]). However, the catalytic zinc appears to coordinate with only three residues: H822, C850 and C853 (Fig. 3b). In DYW1 and OTP86, the catalytically important glutamate residues E173 and E894, respectively, bind a water molecule that acts as the fourth coordinating ligand for the catalytic zinc ion (Takenaka et al. 2021). Based on our modeling, there is no obvious equivalent residue either at a similar position in NbIPI1 as these critical glutamate sidechains. Nor is there a different residue that could directly substitute for the coordinating water molecule observed in the OTP86 DYW domain structure. We therefore suggest that NbIPI1 and its orthologs likely do not play a direct role in catalyzing C-to-U deamination, at least using the mechanism described for OTP86 (Takenaka et al. 2021).

### Identification of predicted NbIPI1 targets

**Fig. 4:**
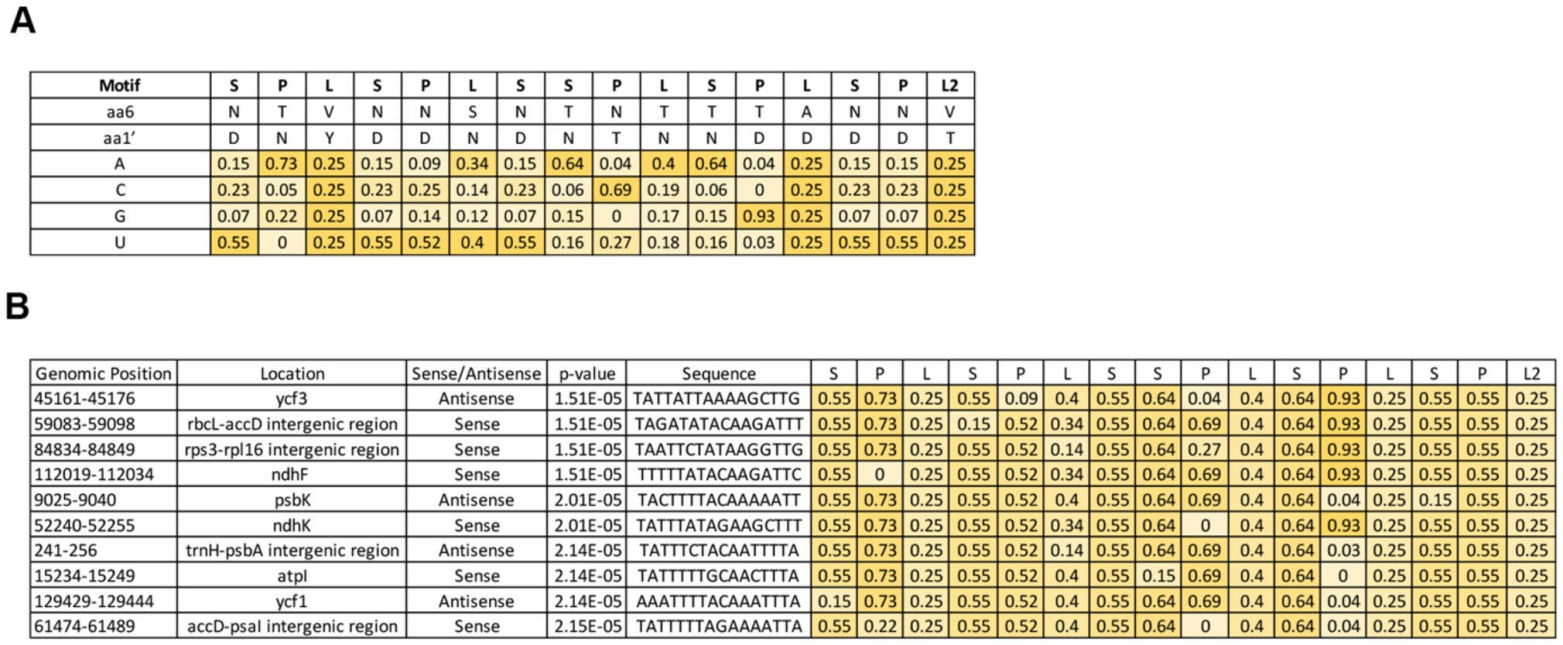
PPR code-based prediction of NbIPI1 binding sites. **a** Nucleotide-binding probabilities for NbIPI1 motifs (P, L, and S) based on the amino acids found at the 6 and 1′ position (first amino acid of the subsequent C terminal PPR motif) of each PPR motif (see Supplementary Figure S1). **b** Prediction of NbIPI1 binding sites within *N. benthamiana* chloroplast genome. The ten top-ranking matches are shown. The genomic location and nucleotide sequence of each site are indicated, along with the binding score for each repeat. The *P-*values were calculated with the FIMO program

PPR proteins bind their RNA targets in a combinatorial manner, using residues from adjacent PPR modules to identify specific RNA bases (Barkan et al. 2012). We used publicly available software tools for prediction of PPR-binding sequences (Barkan et al. 2012; Takenaka et al. 2013; Kobayashi et al. 2019) to identify potential NbIPI1 targets. Computationally predicted sites for Arabidopsis IPI1/EMB175 included an *rps3-rps16* intergenic region that is conserved in maize (Hammani et al. 2016). Supporting the value of computationally predictions in revealing the action of a PPR protein, maize PPR103 bound to and stabilized the 5’ end of the processed maize *rps16* mRNA. A refined algorithm suggested that the mitochondrial *NAD2* transcript was the most likely target for AtIPI1 (Kobayashi et al. 2019). However, this finding is not likely relevant to EMB175/IPI1/PPR103 function since IPI1 is likely localized to chloroplasts and not in mitochondria, at least under our experimental conditions (Fig. 1). We therefore focused on identifying potential chloroplast targets for NbIPI1 (Fig. 4a) using the FIMO tool, as previously described (Takenaka et al. 2013; Grant et al. 2011). We identified several potential target sites including sequences within the *rps3-rpl16* intergenic region, like for maize PPR103 (Fig. 4b).

### NbIPI1 is necessary for the accumulation of chloroplast rRNA species

**Fig. 5:**
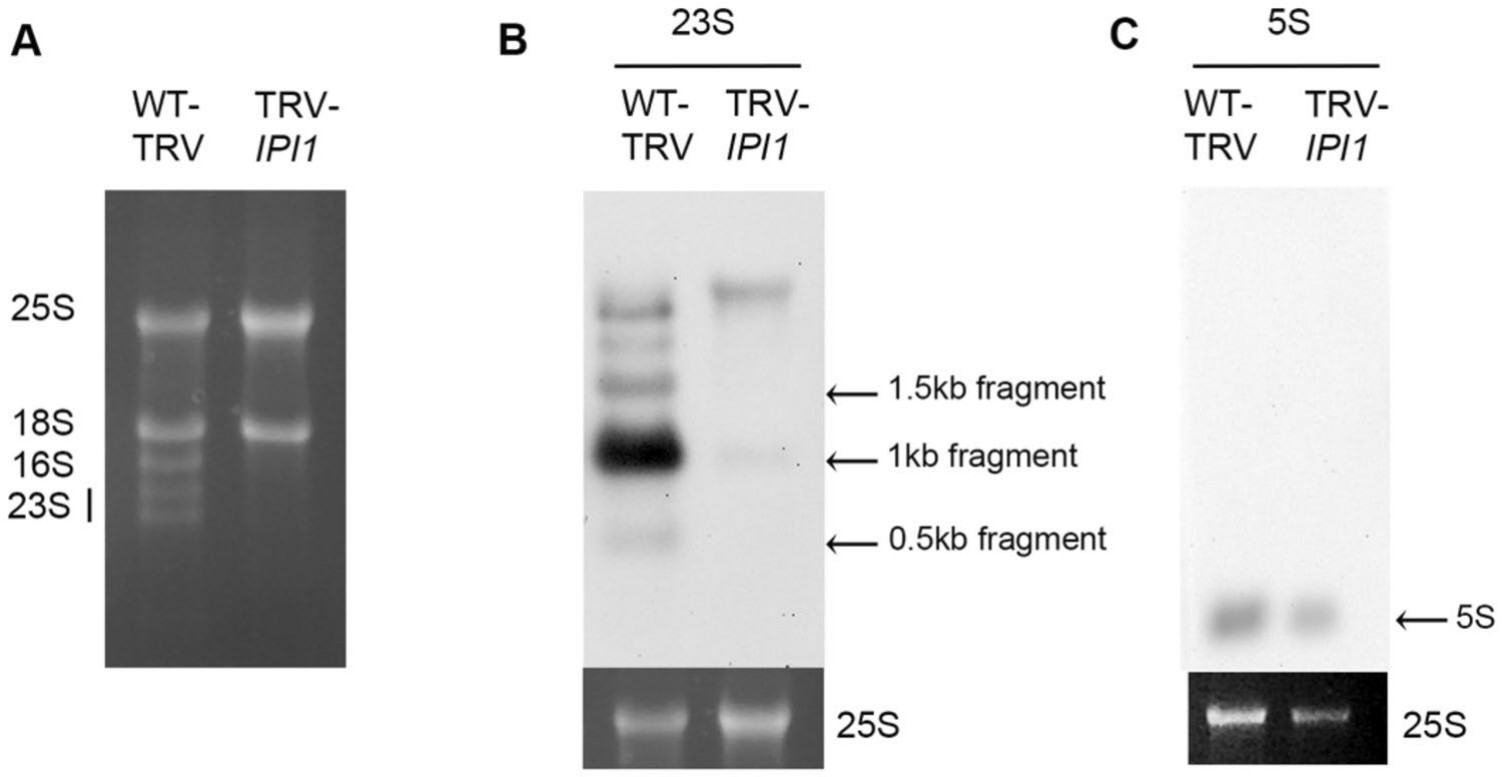
The effects of knockdown of *NbIPI1* expression on rRNA transcripts. **a** One microgram of total RNA from TRV-infected, non-silenced plants and *NbIPI1*-silenced plants stained with ethidium bromide. **b** Northern blot for 23S rRNA. Loading control (bottom panel) shown is from (**a**). **c** Northern blot for 5S rRNA and loading control (bottom panel). All contents of blots are shown, except for the loading controls

Knockdown of *NbIPI1* expression by TRV-mediated VIGS led to severe leaf chlorosis (Fig. 1o) and reduced chlorophyll content and photosystem II quantum efficiency (Ganusova et al. 2020). Similarly, maize *ppr103* mutant seedlings had albino leaves and did not continue development past the seedling stage. Mutant seedlings contained drastically reduced chloroplast ribosomal RNA (rRNA) levels in the *ppr103* albino leaves (Hammani et al. 2016). To test whether NbIPI1 may have a similar role in chloroplast rRNA biogenesis, we performed Northern blotting analysis for chloroplast rRNAs in *NbIPI1*-silenced *N. benthamiana* leaves using sequence-specific probes (Table S1). Defects in rRNA levels were severe enough to be observed on an agarose gel stained with ethidium bromide (Fig. 5a). The Northern blots revealed that silencing *NbIPI1* led to major defects in chloroplast rRNA, with drastic reductions in the 23S rRNA compared to the rRNA levels in the TRV-containing non-silenced controls (Fig. 5b) although there was no obvious change in 5S rRNAs (Fig. 5c). These results suggest that IPI1’s role in rRNA processing is conserved in maize and *N. benthamiana,* although ribosome-associated transcripts were not among the top hits for NbIPI1 targets in our computational analysis (Fig. 4).

## NbIPI1 is required for stability of Nbrpl16 transcripts

**Fig. 6:**
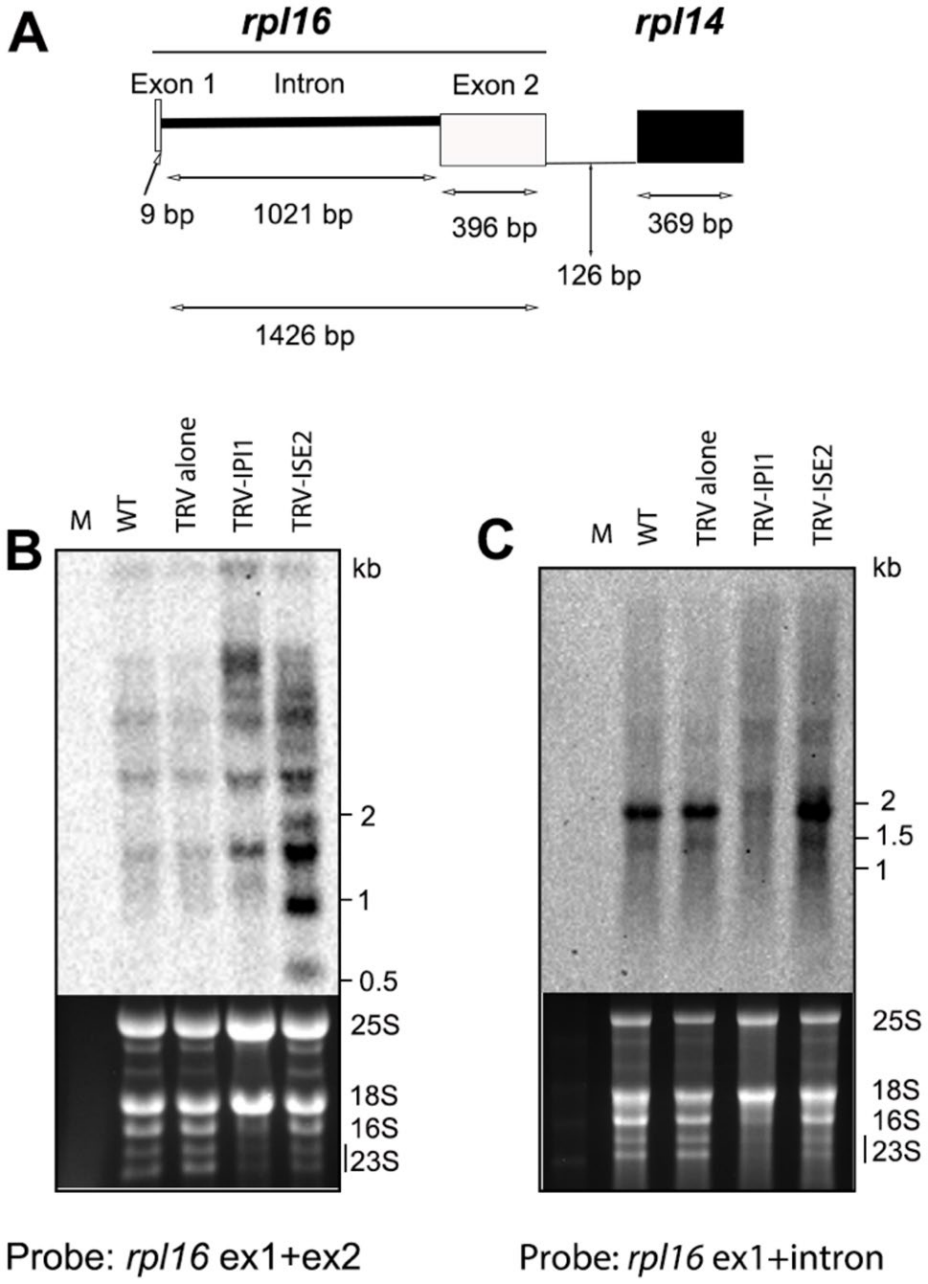
Silencing of *NbIPI1* affects the *rpl16* transcripts. **a** The diagram shows the *rpl16* gene containing two exons (exon 1 is 9 bp and exon 2 is 374 bp) and an intron (1020 bp). RNA from *NbIPI1-* and *NbISE2-* silenced plants were probed with *rpl16* ex1 + ex2 (exon 1 and exon 2) **(b**) and *rpl16* exon1 and intron (**c**), are compared to wild type (WT) and non-silenced control (TRV alone). 4 µg and 2 µg of total RNA were loaded for (**b**) and (**c**), respectively

The *rps3-rps16* intergenic region was one of the top 10 predicted targets for NbIPI1 (Fig. 4b), similar to maize PPR103. PPR103 binding to this intergenic region was found to be required for specification of the 5’end of *rpl16* transcripts (Hammani et al. 2016). The role of NbIPI1 in processing of *Nbrpl16* transcripts was therefore examined. RNA gel blot hybridization using a probe for the *rpl16* exons only (Fig. 6a) revealed a marked accumulation of unprocessed transcripts of higher molecular weights, 3 to > 6 kb but did not detect the expected processed transcripts of 1.4 and 0.4 kb (Fig. 6A and B). However, when probes against the *rpl16* intron were used, the 1.4-kb species representing the full-length transcript and the 1.9 kb bicistronic *rpl16-rpl14* transcript were absent in *NbIPI1*-silenced tissues compared to TRV-only infected and uninfected control (WT) plants (Fig. 6c). Importantly, silencing *NbISE2* did not change the stability of these RNA species (Fig. 6c), suggesting that NbIPI1 is required for processing the *Nbrpl16* transcript and for determining its stability. This is consistent with the function of PPR103 in processing maize rpl16 transcripts and suggests a conserved function for NbIPI1.

### NbIPI1 functions in chloroplast C-to-U RNA editing in N. benthamiana

**Fig. 7:**
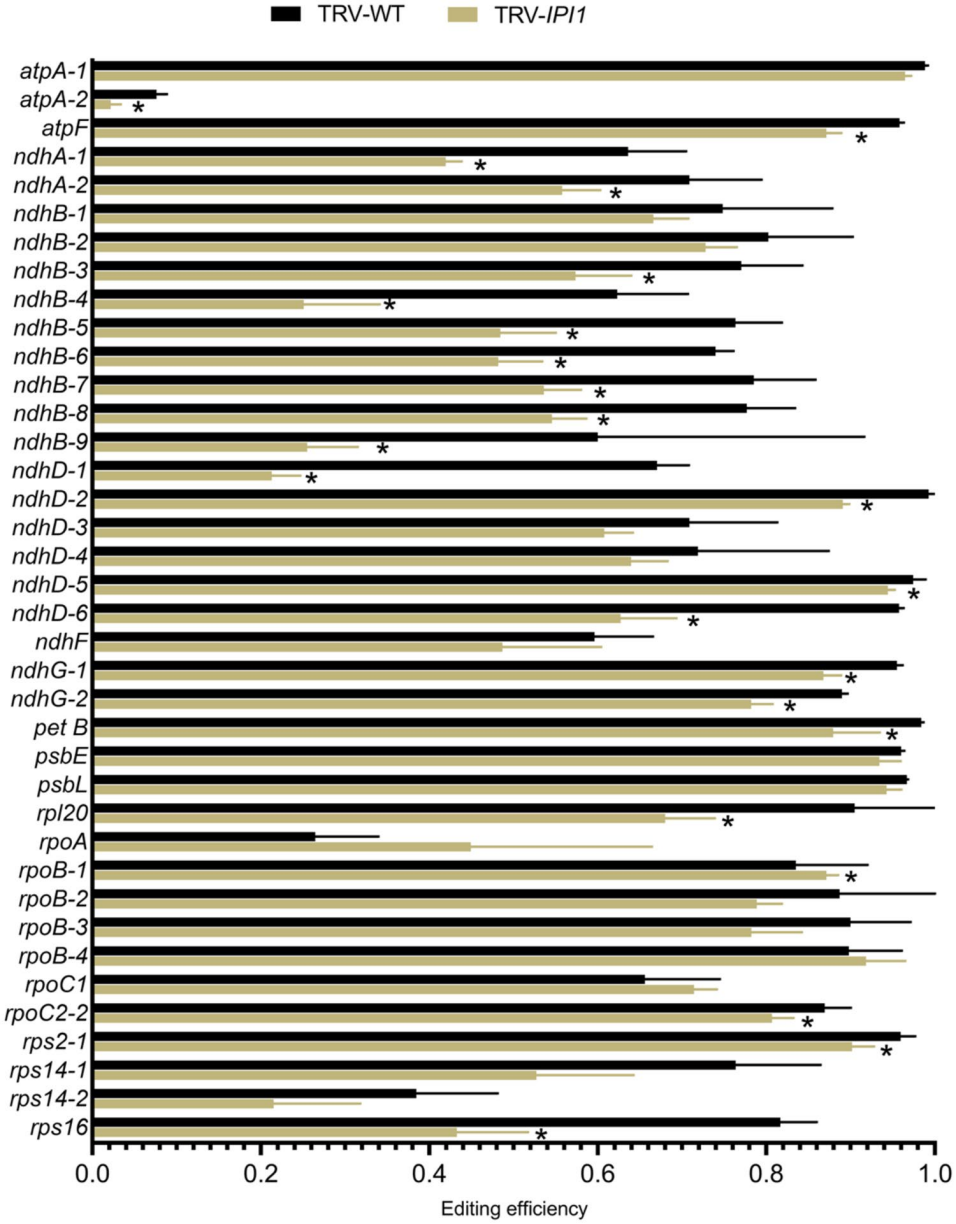
C-to-U editing in *IPI1*-silenced plants. RNA-seq analysis of *N. benthamiana* chloroplast transcripts confirms editing of all sites identified by Sanger sequencing and reveals reduced editing of several sites in *NbIPI1*-silenced chloroplasts. Editing efficiency was calculated by number of reads carrying the edited base as a proportion of the total number of reads for a given site. Error bars represent standard deviation of reads from individual libraries, with four replicates used for *NbIPI1*-silenced chloroplasts and three for the TRV-infected non-silenced controls. Asterisks denote *P*-value < 0.05 as determined using a one-tailed *t*-test assuming unequal variance

Given that NbIPI1 appeared to have distinct functions from maize PPR103, we examined its role in RNA editing. RNAseq analysis was conducted on total RNA from chloroplasts from leaves of *NbIPI1*-silenced or non-silenced control *N. benthamiana* plants. To ensure that enough chloroplasts were available for RNA isolation and subsequent cDNA library preparation, we pooled all chlorotic leaves from one silenced plant to produce one biological replicate. Samples from TRV-infected non-silenced control plants were similarly generated. The chloroplasts from control plants were phenotypically normal as observed by bright-field microscopy, whereas almost all chloroplasts isolated from *IPI1-*silenced mutant tissue were distinctively defective, with no thylakoid structures or starch granules apparent (Supplementary Figure S3), as expected from TEM observations (Fig. 1). Three control RNA-seq libraries (TRV-infected, non-silenced) and four test libraries (VIGS-*IPI1*) were used for Illumina MiSeq analysis (see Materials and Methods and Supplementary Figure S5). The reads had high quality mapping scores before and after the adapters were trimmed, ensuring that good quality contigs were subsequently mapped to the *N. benthamiana* chloroplast reference genome. We mapped the 250-nucleotide long sequenced reads to the *N. benthamiana* chloroplast reference genome curated by the Queensland University of Technology (https://sefapps02.qut.edu.au/benWeb/subpages/chloroplast.php) with no mismatch penalty to allow the detection of multiple editing events within the same transcript. Editing events were detected using an embedded SNP detection algorithm in the DNA Array Star workflow and are represented as the fraction of reads with an edited base out of the total reads (edited + unedited) for a given site. Additionally, reads that mapped to multiple locations were not excluded from subsequent analysis to allow the detection of potential SNPs on the inverted repeat strand of *ndhB*, a transcript that is heavily edited (Fig. 7). The editing efficiency at 23 edited sites in 18 chloroplast transcripts was statistically significantly reduced in *IPI1-*silenced leaves compared to control leaves (Fig. 7). This result suggested that reduced levels of NbIPI1 impacted editing of multiple chloroplast transcripts.

### NbIPI1 may have roles in editing specific transcripts

**Fig. 8:**
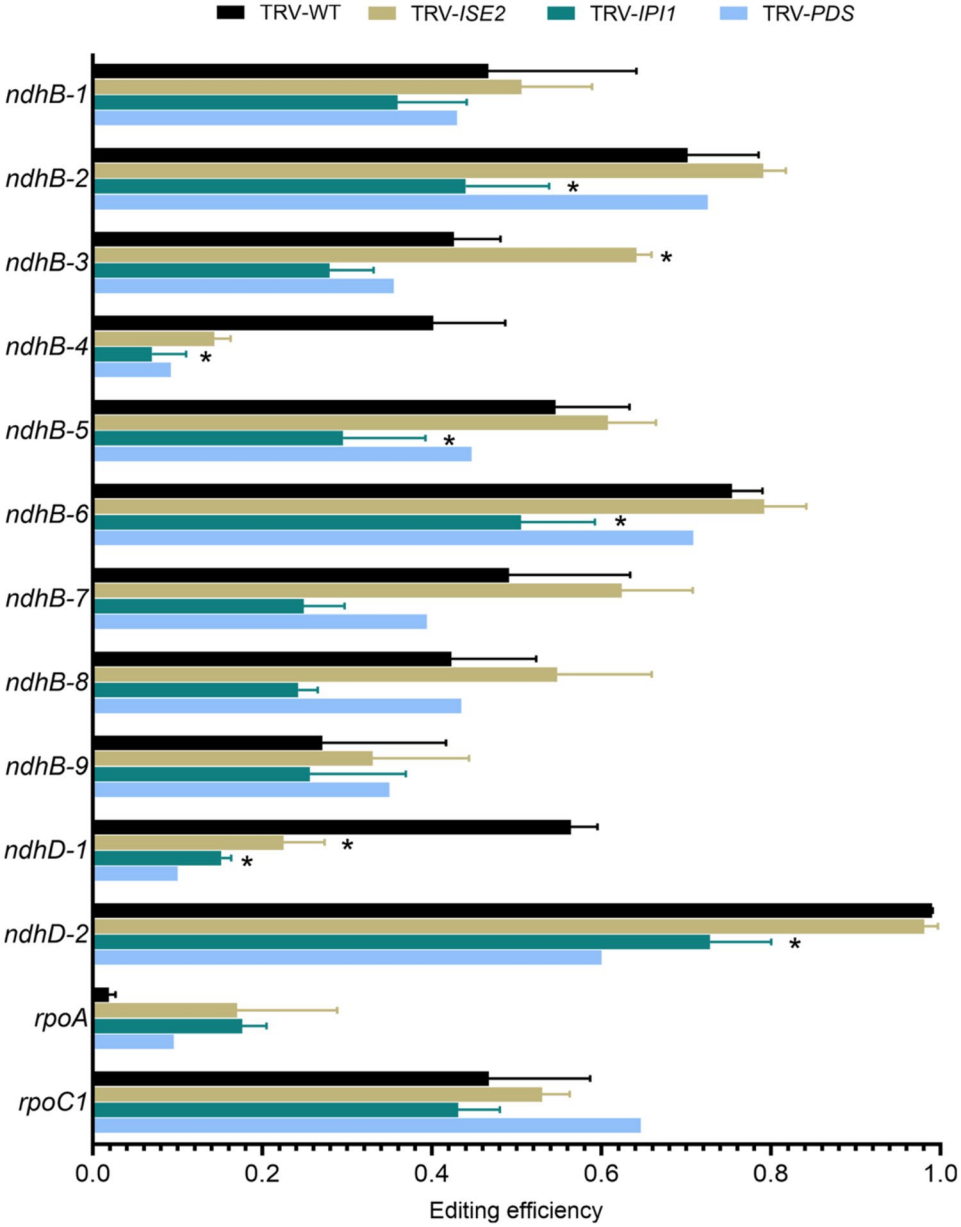
Effects of knockdown of *NbIPI* or *NbISE2* on editing efficiency. RNA editing compared between *N. benthamiana* plants displaying chlorosis resulting from silencing *IPI1*, *ISE2* or *PDS* and TRV-infected, non-silenced controls by Sanger sequencing of transcripts. Results represent two non-silencing control biological replicates, three biological replicates for silencing *IPI1* or *ISE2*, and one biological replicate for silencing *PDS*. Error bars represent standard deviation for each replicate. Asterisks denote *P*-value < 0.05 as determined using a one-tailed Student’s *t*-test assuming unequal variance, pairwise comparisons were made between controls and silenced plants

Since AtIPI1/EMB175 interacts with AtISE2 which was previously shown to be required for chloroplast C-to-U editing, it is possible that some defects in editing *IPI1*-silenced chloroplasts were due to indirect effects on ISE2 activity. In addition, general stress in chlorotic leaves is also known to have deleterious effects on C-to-U editing (Kakizaki et al. 2009; Tseng et al. 2013; Zhu et al. 2014). For these reasons and to confirm the results from the deep sequencing approach, we bulk sequenced selected chloroplast transcripts from *IPI1*-, *ISE2*- or *PHYTOENE DESATURASE* (*PDS*)-silenced plants and compared the results to editing in non-silenced TRV-infected control plants. Sanger sequencing confirmed reduced editing in transcripts from *IPI1*-silenced leaves (Fig. 8). As expected, knockdown of *ISE2* or *PDS* in silenced plants also resulted in defective C-to-U editing (Fig. 8). The defective editing of the *rpoA, ndhB-4*, and *ndhD-1* sites is likely a secondary effect of chloroplast dysfunction since these sites also had editing defects in other chlorotic leaves (*PDS*-silenced). Closer examination of editing defects at other sites revealed that the editing “signatures” caused by knockdown of *IPI1* or *ISE2* are distinct (Fig. 8). For example, the *ndhB-3* site was uniquely affected in *ISE2*-silenced plants and showed a surprising drastic increase in editing when *ISE2* expression was knocked down, while the *ndhB-5* and *-6* sites were uniquely affected in *IPI1*-silenced plants (Figs. 7 and 8). This result suggests that while some editing defects may be due to a general stress response, *NbIPI1* may also be involved in C-to-U editing of a subset of chloroplast transcripts.

## Discussion

Chloroplast development and function are essential for plant survival and, perhaps not surprisingly, mutants with defects in chloroplast often fail to complete embryogenesis or live past the seedling stage (Asakura et al. 2004; Bryant et al. 2011). Correct RNA processing for chloroplast gene expression is a critical aspect of chloroplast development, and much has been learnt about RNA metabolism in chloroplasts (Maier et al. 2008; Stern et al. 2010; Wang et al. 2021). The results presented herein support a role for NbIPI1/PPR103 in rRNA processing, previously reported for its maize orthologue PPR103 (Hammani et al. 2016). We find that NbIPI1 is involved in the processing of rRNA transcripts (Figs. 5 and 6). The results also extend NbIPI1’s possible role to C-to-U editing of chloroplast transcripts (Figs. 7 and 8). Our analysis of RNA editing in *NbPDS*-silenced leaves suggests that NbIPI1 may contribute to editing three sites within the *ndhB* transcript (Fig. 8). Most DYW PPR proteins have a single editing target and little redundancy between editing factors has been observed, probably due to the specificity with which each protein binds its target RNA (Barkan and Small 2014; Gutmann et al. 2020). Indeed, at least six PPR proteins have been identified as required for editing the Arabidopsis *ndhB* transcript, with each PPR protein targeting a single site (Hammani et al. 2009; Okuda et al. 2009, 2010; Hayes et al. 2013; Fu et al. 2022;). In contrast, Arabidopsis QED1 edits one site in each of five Arabidopsis chloroplast transcripts, an usually large number of sites for a single PPR protein (Wagoner et al. 2015). NbIPI1 is potentially involved in editing at least two sites in the *ndhB* transcript from *N. benthamiana* chloroplasts; however, further analysis is required to determine whether these sites and others in edited transcripts (Fig. 7) are specific NbIPI1 targets. Indeed, the computational prediction of targets suggests that editing of other transcripts could potentially involve NbIPI1 (Fig. 4).

### The role of the DYW domain in editing

Nucleus-encoded RNA processing factors that are organelle-targeted are responsible for RNA editing and consistent with this, defects in chloroplast translation do not affect RNA editing (Zeltz et al. 1993). Thus far, nuclear-encoded PPR proteins have been classified as site-specific trans-factors involved in RNA editing (Mach 2009). The DYW domain’s predicted structure resembles that of cytidine deaminases which bind zinc as part of their catalytic activity (Salone et al. 2007; Iyer et al. 2011). Mutations within the DYW domain of DYW1, a PPR protein that is similar to IPI1, greatly impair both zinc-binding and RNA editing, indicating that the DYW domain may confer cytidine deaminase activity (Okuda et al. 2009). Interestingly, the maize IPI1 orthologue PPR103 does not have the specific C-terminal amino acid residues DYW or any variation thereof are not at its C-terminus (Supplementary Fig. S2), and no editing events attributed to PPR103 were disrupted in the maize *ppr103* mutant (Hammani et al. 2016). This observation suggests that the specific DYW triad motif or its variants contribute to the editing reaction at several chloroplast editing sites. Several recent studies have clarified the importance of the DYW domain in RNA editing. Heterologous expression of a moss (*Physcomitrium patens*) PPR protein, PPR65, in *E. coli* was sufficient to edit the co-expressed transcript of the corresponding mitochondrial site, ccmFCeU103PS, with 70–100% efficiency (Oldenkott et al. 2019). A second moss PPR protein, PPR56, was also able to edit both its targets, nad4eU272SL and nad3eU230SL in the *E. coli* system with similar efficiencies as those observed *in planta*. In vitro assays demonstrated that purified recombinant moss PPR65 could successfully perform C-to-U editing of its synthetically generated target RNA, and that the editing activity required zinc and was enhanced by ATP or nonhydrolyzable nucleotide analogs (Hayes and Santibanez 2020). Together these studies advanced the idea that DYW proteins can independently carry out C-to-U editing.

The mechanism of the DYW domain has been more fully revealed by details gleaned from recent crystal structures of the DYW domain of *Arabidopsis thaliana* OTP86 protein, which specifically edits a site in the *rps14* transcript (Takenaka et al. 2021). These studies revealed a cytidine deaminase fold and a DYW domain containing zinc atoms that were critical to editing activity through the regulation of a “gated zinc shutter”. In vitro RNA editing assays confirmed the importance of highly conserved residues to catalysis and highlighted the importance of the coordinating Zn ions for catalysis (Zn1) or stability of the DYW motif (Zn2) (Takenaka et al. 2021), supporting findings from previous mutational studies with other DYW proteins (Hayes et al. 2013, 2015; Boussardon et al. 2014; Oldenkott et al. 2019; Hayes and Santibanez 2020). Our data support the importance of the DYW domain for editing as they demonstrate that a PPR103 family member carrying the DYW triad motif may contribute to editing (Figs. 7 and 8). However, As NbIPI1 and *Arabidopsis* EMB175 family members are missing a critical glutamic acid residue that is intimately involved in the proposed “gated zinc shutter” mechanism, it is not currently clear whether NbIPI1 can edit its target RNAs by itself. We suggest that PPR/variant DYW proteins such as NbIPI1 may a) be catalytically active through a mechanism that is distinct from that of canonical DYW proteins such as OTP86; or b) may not be catalytically active themselves but may function as a specific scaffold or adaptor for a catalytically active partner. The latter could include other PPR proteins within editosome complexes.

Our analysis does not rule out the possibility that the observed reductions in editing in *NbIPI1*-silenced plants are a secondary effect of defective ribosome assembly and the resulting chlorosis. However, editing at some sites, *ndhB-5* and *-6* for example, was only affected in the *NbIPI1*-silenced plants (Figs. 7 and 8). It is typical that loss of catalytic RNA editing factors will result in a complete abrogation of editing of their target editing sites, but there was only about a 30–50% reduction in editing efficiency of *ndhB-5* and *-6* in the silenced plants. It is possible that this reduction (and not loss) was due to the VIGS assay causing a reduction of NbIPI1 levels and not total loss of the protein combined with the heterogeneity of VIGS. Further, if NbIPI1 is not the catalytic protein involved in editing a transcript but instead is playing a supporting role in the context of a larger complex, it is possible that there would be remnant editing activity when NbIPI1 levels are reduced.

### NbISE2 and NbIP1 function in RNA editing in N. benthamiana

ISE2 is an evolutionarily conserved chloroplast-localized RNA helicase that has many roles in RNA processing (Carlotto et al. 2016; Bobik et al. 2017). ISE2 is required for C-to-U RNA editing at multiple sites in Arabidopsis (Bobik et al. 2017), and the maize ortholog was identified in multi-protein complexes that edited the C473 site in maize *ndhA* transcripts (Sandoval et al. 2019). The ISE2-containing editing complex from maize also contained the non-PPR editing factors RIP1/MORF8 and RIP9 as well as ORRM, containing an RNA-Recognition Motif (RRM) and OZ1, a RanBP2-type Zn finger protein. Several PPR proteins including six P-type PPR proteins previously not known to be involved with RNA editing were also identified (Sandoval et al. 2019). The interaction of ISE2 with other editing factors and PPR proteins is consistent with the identification of several RNA binding proteins including IPI1 as interacting with ISE2 in a yeast two-hybrid screen (Bobik et al. 2019). Reduced ISE2 levels in Arabidopsis led to significantly reduced editing specifically at three sites (*rpoB-338*, *rpoB-551* and *rps14-149*) (Bobik et al. 2017). Interestingly, none of these were among the top predicted targets for NbIPI1 binding (Fig. 4) and the editing of the equivalent sites was unaffected in *NbIPI1*-silenced leaves although there was a small but statistically significant increase for the *rpoB-1* (Arabidopsis *rpoB-338*) site (Fig. 7). Perhaps IPI1 and ISE2 may function as part of an editing complex for only some sites, although this remains to be tested experimentally. It is also possible that IPI1 and ISE2 may function as part of a dynamic editing complex for some sites dependent upon plant developmental stage or tissue type. The composition of the possible NbIPI1-containing editosome should be the focus of future investigations as such information will reveal more about chloroplast RNA editing and the molecular machineries involved.

### Supplementary Information

Below is the link to the electronic supplementary material.

Supplementary file1 (DOCX 18594 kb)

## Data Availability

The dataset supporting the conclusions of this article is available in the NCBI Sequence Read Archive (SRA) repository, PRJNA826083 and https://www.ncbi.nlm.nih.gov/bioproject/PRJNA826083.
