# Supplementary material for: The dicot homolog of maize PPR103 carries a C-terminal DYW domain and may have a role in C-to-U editing of some chloroplast RNA transcripts
Source: Plant Mol Biol. 2024 Mar 15;114(2):28. doi: 10.1007/s11103-024-01424-1 (PMC10940495; doi:10.1007/s11103-024-01424-1)
Supplement: Supplementary file 1 — Supplementary file1 (DOCX 18594 kb) [file 11103_2024_1424_MOESM1_ESM.docx]

Title: The dicot homolog of maize PPR103 carries a C-terminal DYW domain and is required for C-to-U editing of chloroplast RNA transcripts

**Journal Name: Plant Molecular Biology**

**Tyra N. McCray,^a,b^ Mohammad F. Azim^b,c^ and Tessa M. Burch-Smith^a,b,c,1^**

^a^School of Genome Science and Technology, University of Tennessee, Knoxville, TN 37996

^b^Department of Biochemistry and Cellular & Molecular Biology, University of Tennessee, Knoxville, TN 37996

^c^Donald Danforth Plant Science Center, St. Louis, MO 63132

^1^Corresponding author e-mail: tburch-smith@danforthcenter.org

**Supplementary Information**

Figure S1. Amino acid alignment of maize (*Zea mays*) PPR103, Arabidopsis IPI1 and *N. benthamiana* IPI1 proteins.

Figure S2. Phylogenetic tree of PPR103/EMB175 orthologs.

Figure S3. NbIPI1 predicted structure modeled on AtDYW1 and AtOTP86 crystal structures.

Figure S4. Coordination of the structural zinc atom in AtDYW1, AtOTP86 and NbIPI1.

Figure S5. Workflow for sample preparation for RNAseq and appearance of isolated chloroplasts.

Table S1. Primers used in this study.

Table S2. Summary of read quality from RNAseq analysis of chloroplast RNA.

**Supplementary Figure S1**

*Zea mays* MNGHLHLIPSTPPRLHLTSLSPTFLSFPPAPCPHPPPHGPHPRLPLRS------------ 48

*Arabidosis thaliana* MS---------------TVNHHCLLNFPHIPPSIPPNHRPKLLSSLSLYR-KPERLFALS 44

*Nicotiana benthamiana* MA---------------AVIHSPTI---SIPLSFLPPTRPFYLHSTSSLATTKKTKFKQK 42

* : : * * *

*Zea mays* ----------------VAVA-------------------AAADPR--------------- 58

*Arabidosis thaliana* A---------SLSLSPATIHECSSSSSSSSSSFDKEETEDIESVIDGFFYLLRLSAQYHD 95

*Nicotiana benthamiana* PSFFLQYPSKSRPFQPLLIPQHFK---------DSIVTVGADTTRIDYANLLRISVRCGD 93

:

***** *** ***

*Zea mays* AAHAAAVKSGAL-GPSSDARTANAVMCAYLRVGRLGDARDVFDWMPARDAASYSALISGY 117

*Arabidosis thaliana* VEVTKAVHASFLKLREEKTRLG**N**ALISTYLKLGFPREAILVFVSLSSP**T**VVSYT**A**LISGF 155

*Nicotiana benthamiana* VELAKIIHSSILKLEEEDIYLK**N**ALIAAYLKLGHLNLAEKVFNSLLSP**D**VVLY**T**AIISAF 153

. : :::. * ... **::.:**::* * ** : : .. *:*:**.:

*** ***

*Zea mays*  ARLAGGSGSVTATVASAELLGRMRLADGLLPTEYTFVGLFTACARRGNPRLGTQVHALAA 177

*Arabidosis thaliana* SRLNLE-------IEALKVFFRMRKAGLVQPNEYTFVAILTACVRVSRFSLGIQIHGLIV 208

*Nicotiana benthamiana* AKSNRQ-------REAYELFREMRDLG-IEP**N**EYTF**V**AILTACIRSLNLELGRQVHGLVV 205

:: : ::: .** . : *.*****.::*** * . ** *:*.* .

*** * * ***

*Zea mays* KSGHSSLLLVANAILGMYVK--CGRFGDAMRAFDGMDRRDVSSWNAVLAGLVELGRHDEA 235

*Arabidosis thaliana* KSGFLNSVFVSNSLMSLYDKDSGSSCDDVLKLFDEIPQRDVASWNTVVSSLVKEGKSHKA 268

*Nicotiana benthamiana* KLGYLS**Y**TYVV**N**ALMGLYSK--GGLLESVILLFNDMPQR**D**IVSW**N**TTISCMVEECMYERA 263

* *. . * *:::.:* * . ..: *: : :**: ***:.:: :*: ..*

*** * * ***

*Zea mays* FEMFGEMRASGNVRADRFSLSALLTAAGEGVGQLQGEAVHALSFKSGLETDLSVGNALIG 295

*Arabidosis thaliana* FDLFYEMNRVEGFGVDSFTLSTLLSSCTDSSVLLRGRELHGRAIRIGLMQELSVNNALIG 328

*Nicotiana benthamiana* FEMYRELRRNDCLIV**D**HFTL**S**MLLAASSRCLAVREGQELHAHALKSGLHG**N**LSVN**N**ALIG 323

*::: *:. . .* *:** **::. .*. :*. ::: ** :***.*****

*** *** *** ***

*Zea mays* FYAEHGASVEDVVSVFQRMPVKDVISWTGLLNGYMEFGLVDMALDVFERMPQRNFVTYNA 355

*Arabidosis thaliana* FYSKFWD-MKKVESLYEMMMAQDAVTFTEMITAYMSFGMVDSAVEIFANVTEKNTITYNA 387

*Nicotiana benthamiana* FYTKCGT-LKNVVDMFERMPVK**D**VFSW**T**EMIVAYMEFGYVDFAMDIFNSMPER**N**CVSY**N**A 382

**:: ::.* .::: * .:*..::* :: .**.** ** *:::* : ::* ::***

*** ***

*Zea mays* VLTGFCRNKEGVRATFAKKAGLRGLGLFRQMVEDGLEISDVTVTGVLNACAIAADRKVSE 415

*Arabidosis thaliana* LMAGFCRNGHGL----------KALKLFTDMLQRGVELTDFSLTSAVDACGLVSEKKVSE 437

*Nicotiana benthamiana* LLAGFTQNHEGF----------KALVLFCRMLEGGMEL**T**DFTL**T**SVLNACGSMTERKISE 432

:::** :* .*. :.* ** *:: *:*::*.::*..::**. :::*:**

*** *** *** ***

*Zea mays* QVHAFVIKCGCVSSPWSDAALIDMCIKCGRSGDAHLLFEQWQHEESFHIAWNSLLLASVR 475

*Arabidosis thaliana* QIHGFCIKFGTAFNPCIQTALLDMCTRCERMADAEEMFDQWPSNLDSSKATTSIIGGYAR 497

*Nicotiana benthamiana* QIHAFILKLGLES**N**DRIE**T**ALLDMCTRCERMDDAKKIFHQLPLDHD**N**SVAL**T**SMMCAYAR 492

*:*.* :* * . ::**:*** :* * **. :*.* : . * .*:: . .*

*** * ***

*Zea mays* GGEYEKALSTFLQMFRSSGAEFIDEFMLTSVLGVCGSLGFAELGKQMHTFAAKSGLLSAR 535

*Arabidosis thaliana* NGLPDKAVSLFHRTL-CEQKLFLDEVSLTLILAVCGTLGFREMGYQIHCYALKAGYFSDI 556

*Nicotiana benthamiana* DGHPEEAISLFLVRH-SEESLVV**D**EVAL**A**TILGICGTLGILKLGEQIHCYALKHGLMS**D**T 551

.* ::*:* * .. .:**. *: :*.:**:**: ::* *:* :* * * :*

***** *** ***

*Zea mays* GVGNAIISMYGKCGELKDAISLFERMSCRDLVSWNAMITAHLLLHQGDDILKIWSEMERS 595

*Arabidopsis thaliana* SLGNSLISMYAKCCDSDDAIKIFNTMREHDVISWNSLISCYILQRNGDEALALWSRMNEK 616

*Nicotiana benthamiana* GVG**N**AMISMYSKCDEMQSAIKAFEAMPTH**D**LVSC**N**GLLTCYVLHRQGDAALNMWAKMENL 611

.:**::****.** : ..**. *: * :*::* *.:::.::* ::** * :*:.*:.

*** * * ***

*Zea mays* MVRPDSITFLLVISACSHTSSDSTDKCRKLFLSMPSTYGIEPAMEHYAAFVYVLGCWGRF 655

*Arabidosis thaliana* EIKPDIITLTLVISAFRYTESNKLSSCRDLFLSMKTIYDIEPTTEHYTAFVRVLGHWGLL 676

*Nicotiana benthamiana* GVKP**DS**ITC**V**LVISAYRHTSRNLVDCCQKFFSSMQSSYKVKP**T**SEHY**A**GFVGVLGYWGLL 671

::** ** ***** :*. : . *:.:* ** : * ::*: ***:.** *** ** :

*****

*Zea mays* DEAEQLIGGMPLQPGALVWRSLLDSCSKHSNMAVRRRAMKHLLALEPQDPSTYVLTSNLL 715

*Arabidosis thaliana* EEAEDTINSMPVQPEVSVLRALLDSCRIHSNTSVAKRVAKLILSTKPETPSEYILKSNIY 736

*Nicotiana benthamiana* EEAEQIISAMPFEP**K**ASVWHALLEGCRIHVNAIIGKRAMKEILSIAPQDPSTFILKSNLY 731

:***: *..**.:* . * ::**:.* * * : :*. * :*: *: ** ::*.**:

*Zea mays* SESARWRSSENTRLEMYEKGMRKIPARSWTFHGNMVHSFFARDKSHPQSRDIYAGLDVLI 775

*Arabidosis thaliana* SASGFWHRSEMIREEMRERGYRKHPAKSWIIHENKIHSFHARDTSHPQEKDIYRGLEILI 796

*Nicotiana benthamiana* SASGRWQCSELVRAEMREKGFRKIPGRSWIILGDKVHSFFARDKLHSQSKDIYSGLQILI 791

* *. *: ** * ** *:* ** *.:** : : :***.***. * *.:*** **::**

*Zea mays* LECIKAGYEPDTTFVLHDVEEYQKRHFLMYHSMKLASMYGLLMA-GPGQTIRVVKNIRMC 834

*Arabidosis thaliana* MECLKVGYEPNTEYVLQEVDEFMKKSFLFHHSAKLAVTYGILSSNTRGKPVRVMKNVMLC 856

*Nicotiana benthamiana* LECLKAGYVPDTSFVLHEVEEHQKKDFLFYHSSKLAVTFGLLMT-RPGKPVRVMKNVHLC 850

:**:*.** *:* :**::*:*. *: **::** *** :*:* : *: :**:**: :*

*Zea mays* GDCHSFLEHASAATGKVISVRDSSGFHIFRGGKCSCSQ-- 872

*Arabidosis thaliana* GDCHEFFKYISVVVKREIVLRDSSGFHHFVNGKCSCRDLW 896

*Nicotiana benthamiana* GDCHTFFKYVSVVTKKDIHIRDASGFHHFVNGKCSCRDNW 890

**** *::: *... : * :**:**** * .***** :

**Supplementary Figure S1**. Multiple sequence alignment of maize *N. benthamiana* PPR103 and orthologous proteins from Arabidopsis and maize (*Zea mays*)*.* PPR motifs are color coded with underlines as follows: S-motifs in yellow, P motif in orange, and L motifs in red. The E and DYW domains at the C-terminus are underlined in gray and blue, respectively. The two amino acids that typically specify the nucleotide bound by PPR motifs (amino acid 6 and 1’) are indicated by red and blue stars, respectively.

**
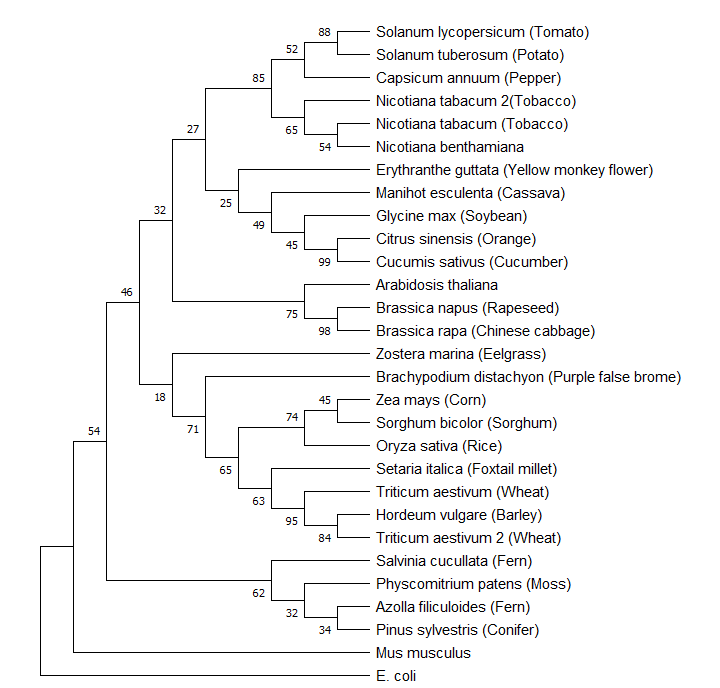
Supplementary Figure S2**

**Supplementary Figure S2.** The phylogenetic tree of PPR103/EMB175 orthologs was generated using the Neighbor-Joining method. The bootstrap values calculated as per million for 1,000 replications are shown at nodes. The evolutionary distances were computed using the p-distance method and are in the units of the number of amino acid differences per site. Monocots are shaded in yellow box.

**Supplementary Figure S3**

**
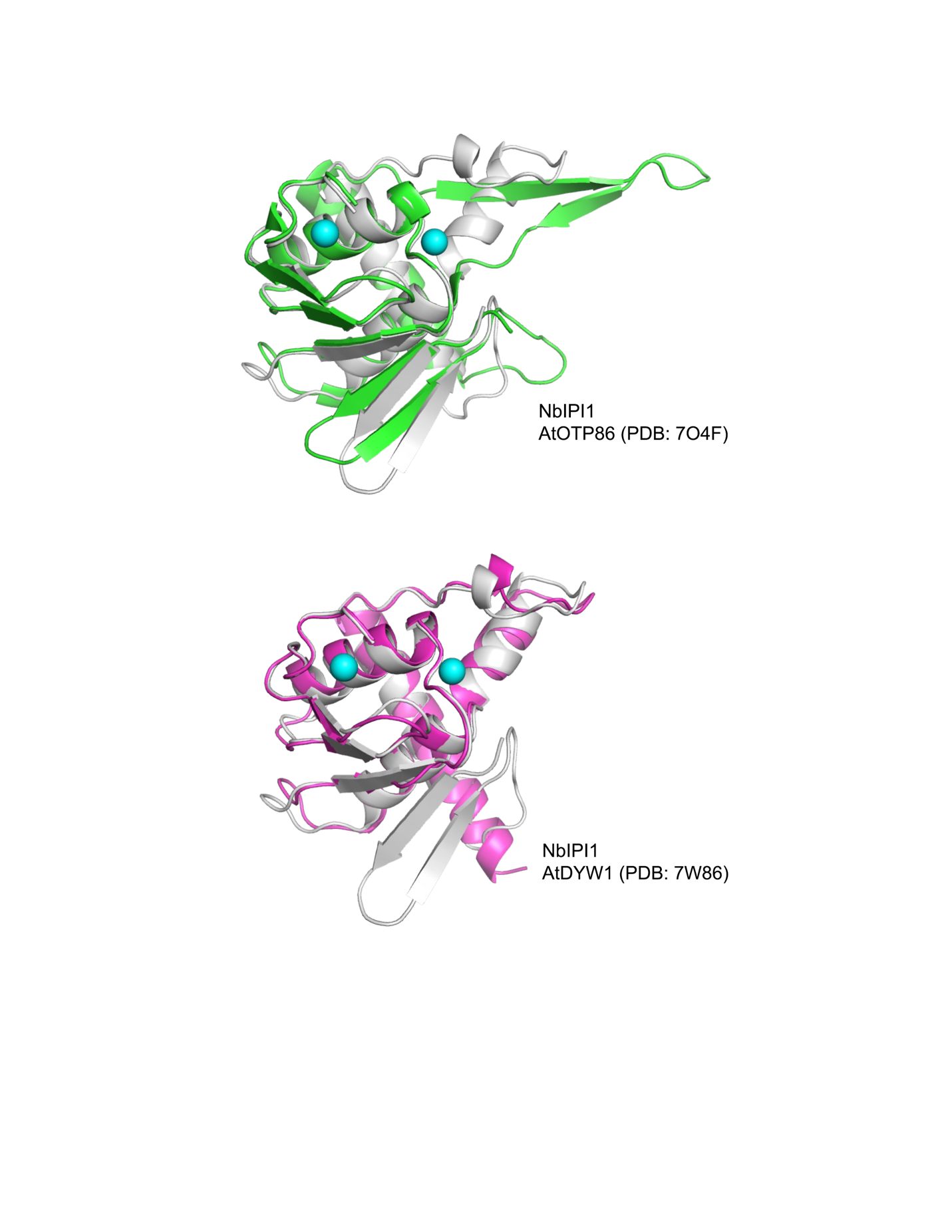
**

**Supplementary Figure S3.** NbIPI1 structure modeled on to that of AtDYW1 (PDB:7W86) or AtOTP86 (PDB:7O4F) using AlphaFold2.

**Supplementary Figure S4**


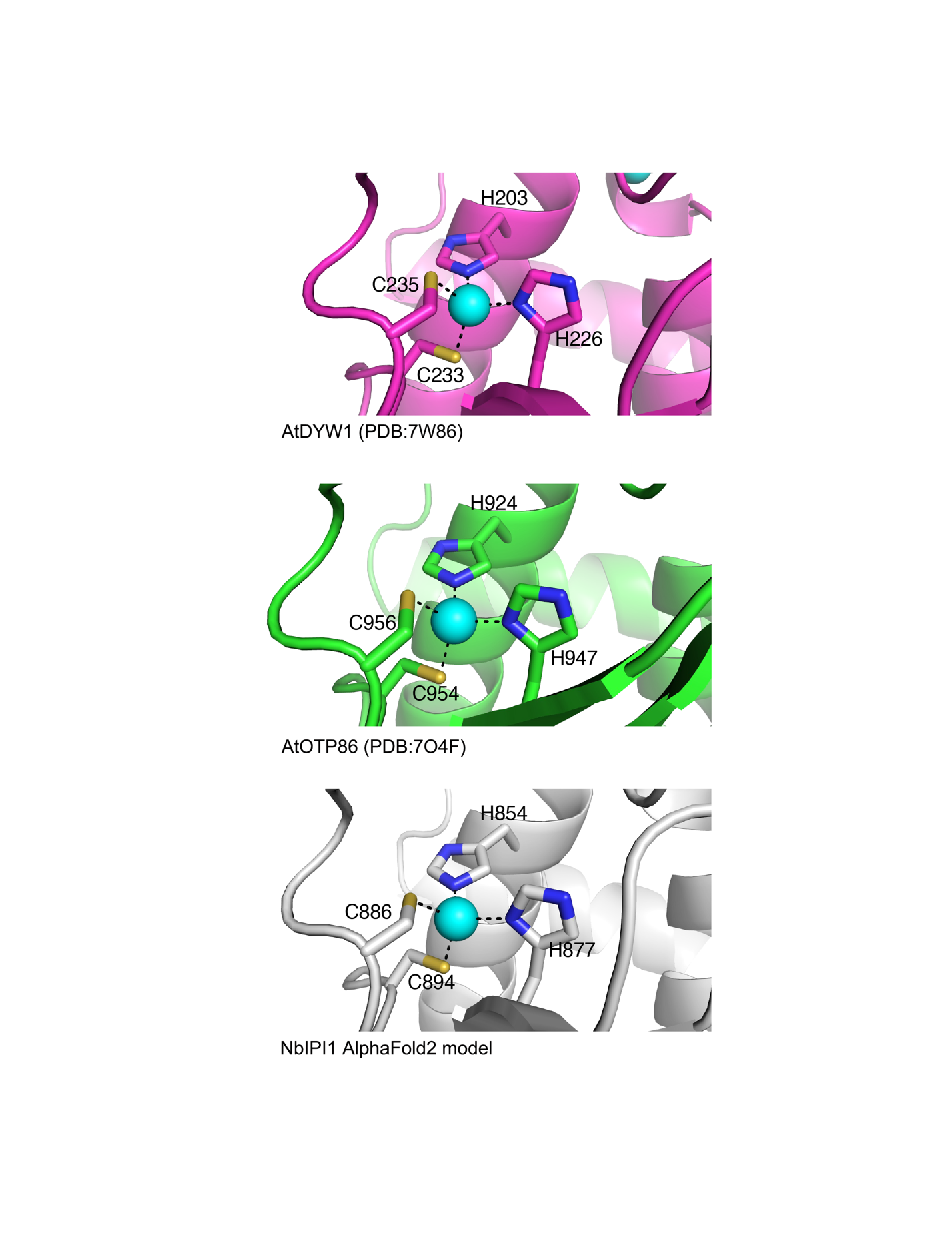


**Supplementary Figure S4.** Closeup view of the structural zinc binding sites in AtDYW1 and AtOTP86 from their crystal structures. Predicted binding of the structural zinc is predicted to be conserved in NbIPI1 by the AlphaFold2 model.

**Supplementary Figure S5**


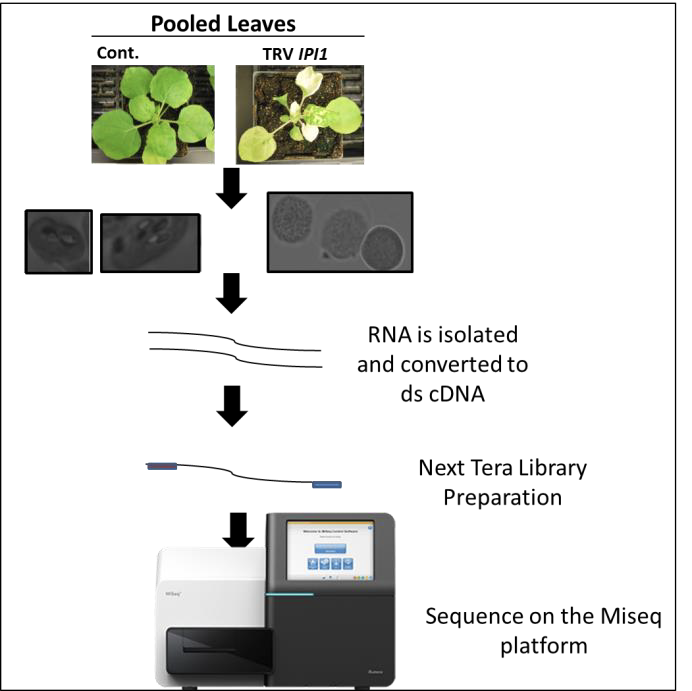


a.

c.

b.

**TRV TRV-*IPI1***


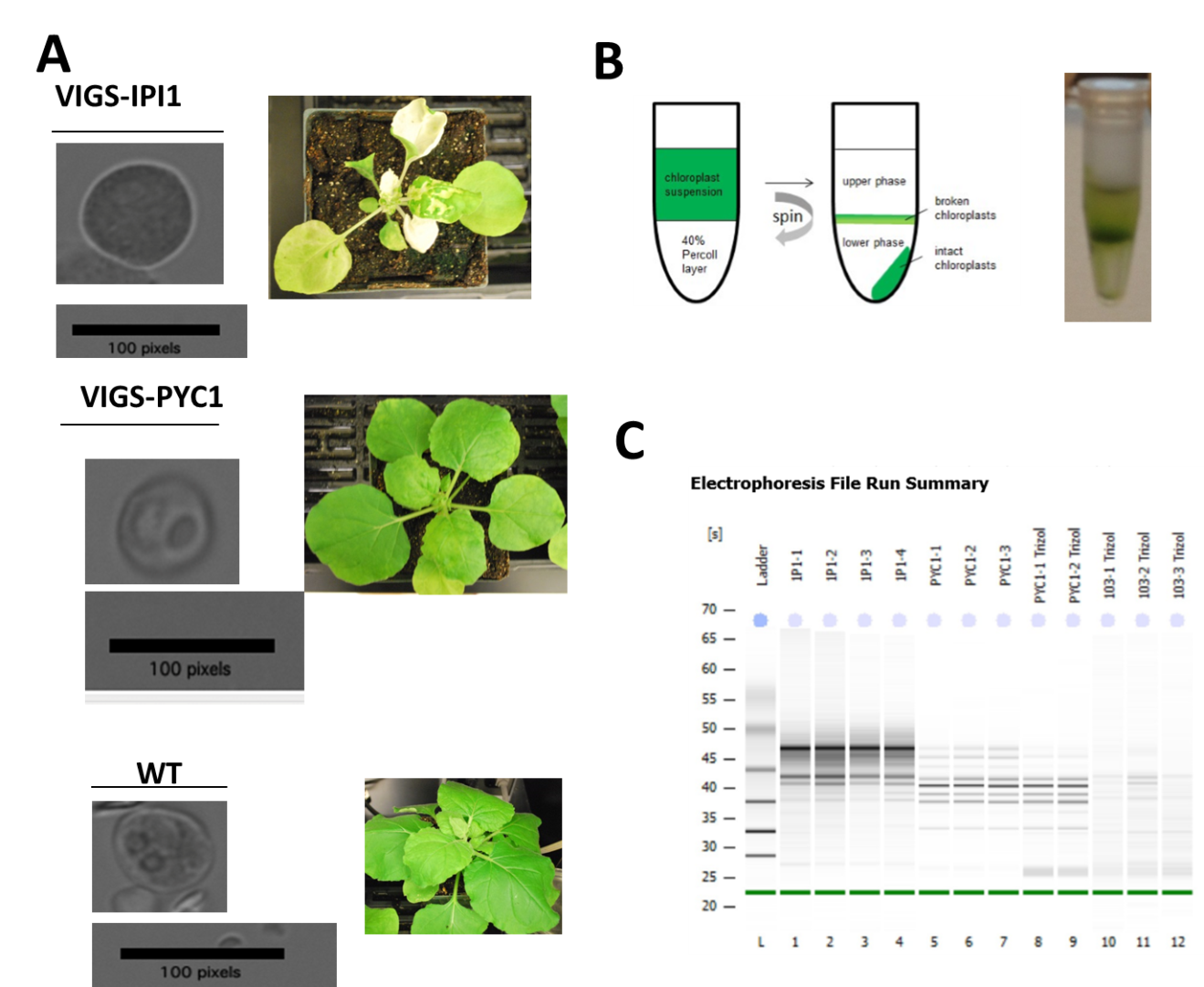

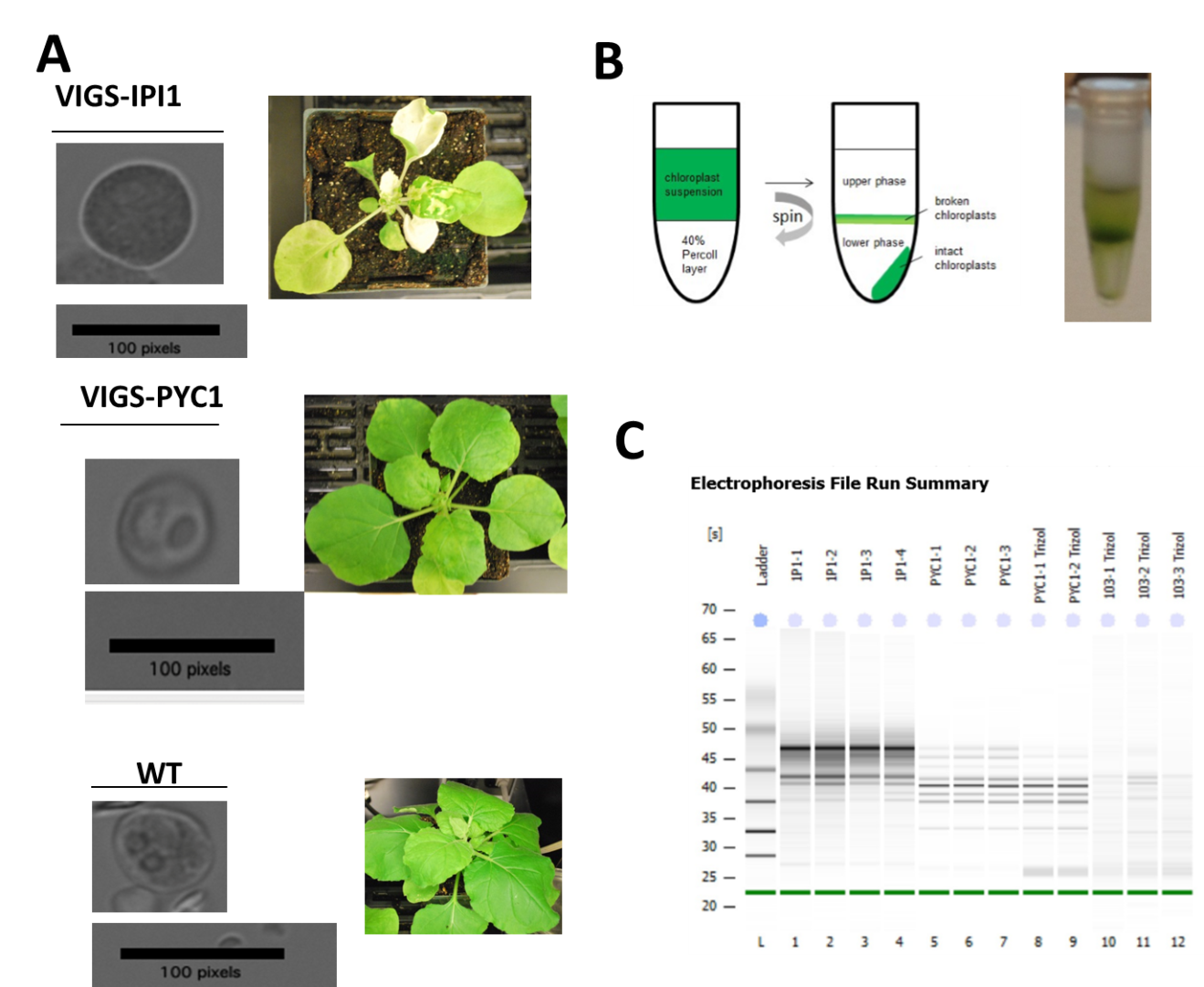


**Supplementary Figure S5**. Workflow for analysis of N. benthamiana chloroplast transcripts by RNAseq. **A.** Leaves were pooled from non-silenced, TRV-infected or *NbIPI1*-silenced plants and chloroplasts were isolated then imaged. RNA was extracted from chloroplasts and then used for cDNA synthesis. After library preparation samples were sequenced on the Illumina MiSeq platform. **B.** Chloroplasts from non-silenced, TRV-infected controls plants contained thylakoid membranes and starch granules. **C.** Chloroplasts from *NbIPI1*-silenced plants did not contain well-developed internal structures or starch granules.

**Table S1. Primers used in this study**

| **Name** | **Oligo Sequence (5’ -> 3’)** | **Purpose** |
| --- | --- | --- |
| *ndhB1-F* | TCTCCCCCGGATGAACCATA | Forward primer to amplify *N. benthamiana* chloroplast transcript containing *ndhB-*1 editing site |
| *ndhB1-R* | TAGTGGATGCTGCCAAAGGG | Reverse primer to amplify *N. benthamiana* chloroplast transcript containing *ndhB-*1 editing site |
| *ndhB 2-6-F* | GATTCGTCGTTCCTGACCCT | Forward primer to amplify *N. benthamiana* chloroplast transcript containing *ndhB-*2-6 editing sites |
| *ndhB 2-6-R* | AGGGGGAATGTTTTTATGCGG | Reverse primer to amplify *N. benthamiana* chloroplast transcript containing *ndhB-*2-6 editing sites |
| *ndhB 7-9-F* | TTCCCATTTTGGGCGGAACA | Forward primer to amplify *N. benthamiana* chloroplast transcript containing *ndhB-*7-9 editing sites |
| *ndhB 7-9-R* | CACTTAGGAGCCGTGTGAGA | Reverse primer to amplify *N. benthamiana* chloroplast transcript containing *ndhB-*7-9 editing sites |
| *ndhD 1,2-F* | ACAGACGTTTCTTTCCTCCCC | Forward primer to amplify *N. benthamiana* chloroplast transcript containing *ndhD*-1,2 editing sites |
| *ndhD 1,2-R* | AGATGTGAATCCGCCTGTCC | Reverse primer to amplify *N. benthamiana* chloroplast transcript containing *ndhD*-1,2 editing sites |
| *rpoA-F* | CCTTTGGTTGGGCATTGGTG | Forward primer to amplify *N. benthamiana* chloroplast transcript containing *rpoA* editing site |
| *rpoA-R* | CCTGTTCGAAACGCGAATCA | Reverse primer to amplify *N. benthamiana* chloroplast transcript containing *rpoA* editing site |
| *rpoC1-F* | TCCACAAGCACAAATTCCGC | Forward primer to amplify *N. benthamiana* chloroplast transcript containing *rpoC1* editing site |
| *rpoC1-R* | AGAAGGGTTTGGGGTTGCTC | Reverse primer to amplify *N. benthamiana* chloroplast transcript containing *rpoC1* editing site |
| *rpl16 ex1+ex2-F* | TGAGTTCGTATAGGCATTTTAG | Forward primer for probe  for Northern blotting that targets exons of *Nbrpl16* |
| *rpl16 ex1+ex2-R* | ACACGTATGGGTTCAGGAAA | Reverse primer for probe for Northern blotting that targets exons of *Nbrpl16* |
| *rpl16 ex1+intron-F* | ACAGAACCGGACGTGAGAGT | Forward primer for probe  for Northern blotting that targets exon1 and intron of *Nbrpl16* |
| *rpl16 ex1+intron-R* | TCCCACGTTCAAGGTATTACTCA | Reverse primer for probe for Northern blotting that targets exon1 and intron of *Nbrpl16* |
| *FL-AtIPI1 Gateway-F* | GGGG ACA AGT TTG TAC AAA AAA GCA GGC TTG ACGACGTTACCGCCATTAGG | attB Forward primer to amplify full-length AtIPI1 |
| *FL-AtIPI1 Gateway-R* | GGGG AC CAC TTT GTA CAA GAA AGC TGG GTT GTCGTTGAGGAACATGCAGAC | attB Reverse primer to amplify full-length AtIPI1 |
| *AtIPI1* TP Gateway-F | GGGGACAAGTTTGTACAAAAAAGCAGGCTatgtccaccgttaatcatcactg | attB Forward primer to amplify the AtIPI1 transit peptide plus codons for ~ 60 nucleotides of the *IPI1* coding sequence to be used for localization experiments |
| *AtIPI1* TP Gateway-R | GGGGACCACTTTGTACAAGAAAGCTGGGTG CCCATCAATAACAGATTCAATGTCTTCCG | attB Reverse primer to amplify the AtIPI1 transit peptide plus codons for ~ 60 nucleotides of the *IPI1* coding sequence to be used for localization experiments |
| *NbIPI1* TP Gateway-F | GGGGACAAGTTTGTACAAAAAAGCAGGCTATGGCTGCCGTTATCCACAGCCCC | attB Forward primer to amplify the *N. benthamiana* transit peptide plus codons for ~ 60 nucleotides of the *IPI1* coding sequence to be used for localization experiments |
| *NbIPI1* TP Gateway-R | GGGGACCACTTTGTACAAGAAAGCTGGGTG ATCACCACACCGAACAGAAATACG | attB Reverse primer to amplify the *N. benthamiana* transit peptide plus codons for ~ 60 nucleotides of the *IPI1* coding sequence to be used for localization experiments |
| Northern 5S-F | TATTCTGGTGTCCTAGGCGTAGAGG | forward for *rrn5S*  Based on chloroplast sequence NC_000932 |
| Northern 5S-R | ATCCTGGCGTCGAGCTATTTTT | reverse for *rrn5S*  Based on chloroplast sequence NC_000932 |
| Northern 23S-F | GAAACTAAGTGGAGGTCCGAACCGAC | 5' for *rrn23S*  IRB23F from Heinz et al. |
| Northern 23S-R | CGCTACCTTAGGACCGTTATAGTTAC | 5' for *rrn23S*  IRB23R from Heinz et al. |

**Table S2. RNAseq mapped reads using SeqMan NGen software.**

|  | **Total Reads Assembled** | | | |
| --- | --- | --- | --- | --- |
|  |  | Single Seq. Count | Split Seq. Count | Seqs. Score =<100% |
| **Control** | 645570 | 9573 | 2319 | 647889 |
| ***IPI1*-silenced** | 559697 | 127037 | 2045 | 561742 |
|  | **Unassem. Seq.** | | | |
|  |  | Unaligned Count | Excessive Cov. Seq. Count | Split Fragments |
| **Control** | 3373 | 3373 | 1336118 | 558 |
| ***IPI1*-silenced** | 2399638 | 10095 | 560214 | 473 |
